# Octanoic Acid—An Insecticidal Metabolite of *Conidiobolus coronatus* (Entomopthorales) That Affects Two Majors Antifungal Protection Systems in *Galleria mellonella* (Lepidoptera): Cuticular Lipids and Hemocytes

**DOI:** 10.3390/ijms23095204

**Published:** 2022-05-06

**Authors:** Agata Kaczmarek, Anna Katarzyna Wrońska, Michalina Kazek, Mieczysława Irena Boguś

**Affiliations:** 1 Witold Stefański Institute of Parasitology, Polish Academy of Sciences, 00-875 Warsaw, Poland; a.wronska@twarda.pan.pl (A.K.W.); m.kamut@twarda.pan.pl (M.K.); slawka@twarda.pan.pl (M.I.B.); 2 Biomibo, 04-872 Warsaw, Poland

**Keywords:** apoptosis, cuticle, free fatty acids, insect hemocyte, oxidative damage to DNA, Sf-9 cell line

## Abstract

The food flavour additive octanoic acid (C8:0) is also a metabolite of the entomopathogenic fungus *Conidiobolus coronatus*, which efficiently infects and rapidly kills *Galleria mellonella*. GC-MS analysis confirmed the presence of C8:0 in insecticidal fraction FR3 extracted from *C. coronatus* filtrate. Topical administration of C8:0 had a dose-dependent effect on survival rates of larvae but not on pupation or adult eclosion times of the survivors. Topically applied C8:0 was more toxic to adults than larvae (LD100 for adults 18.33 ± 2.49 vs. 33.56 ± 2.57 µg/mg of body mass for larvae). The administration of C8:0 on the cuticle of larvae and adults, in amounts corresponding to their LD50 and LD100 doses, had a considerable impact on the two main defense systems engaged in protecting against pathogens, causing serious changes in the developmental-stage-specific profiles of free fatty acids (FFAs) covering the cuticle of larvae and adults and damaging larval hemocytes. In vitro cultures of *G. mellonella* hemocytes, either directly treated with C8:0 or taken from C8:0 treated larvae, revealed deformation of hemocytes, disordered networking, late apoptosis, and necrosis, as well as caspase 1–9 activation and elevation of 8-OHdG level. C8:0 was also confirmed to have a cytotoxic effect on the SF-9 insect cell line, as determined by WST-1 and LDH tests.

## 1. Introduction

The mutual relationship between insects and entomopathogenic fungi is an excellent illustration of a co-evolutionary arms race, characterized by the continuous development of increasingly effective strategies for the exploitation of host insects by attacking fungi and the increasingly strong defense of insects in full accordance with the Red Queen Hypothesis formulated by Van Valen [1].

One such parasite, the cosmopolitan soil fungus *C. coronatus*, was found to induce 90% mortality in important parasites of beekeeping—*G. mellonella* last instar larvae populations exposed to sporulating colonies [2,3,4]. Following exposure, non-specific hydrophobic and electrostatic interactions are formed to join the conidia to the insect cuticle [5]. The growing hyphae then exert pressure on the cuticle and produce cuticle-degrading enzymes to enter the insect body [5,6]. The course of infection depends on cuticle structure and composition, whether any antifungal compounds are present in the exoskeleton, and the cellular and humoral defense reactions of the host [7]. In addition, susceptibility varies between insect species, and this has been attributed to differences in cuticle composition, which affects the germination of the conidia [6,8].

Several metabolites produced by *C. coronatus* demonstrate insecticidal activity for example, coronatin-1 and coronatin-2 [2,9], dodecanol [10], the two β-carboline alkaloids, harman and norharman [11,12] and two trichothecenes, HT-2 and T-2 toxin [13,14,15]. However, research indicates that *C. coronatus* filtrate might be an important source of other entomopathogenic substances.

Octanoic acid (caprylic acid) is a one these metabolites with potential insecticidal activity: It is a saturated fatty acid with the structural formula CH_3_(CH_2_)_6_CO_2_H. It is considered a safe food additive [16] and, in accordance with EU Regulation n°528/2012, it may be used as a safe and non-toxic insecticide against ants (*Lasius niger*), cockroaches (*Blaptica dubia, Blatella germanica, Blatella orientalis, Periplaneta americana*), and crickets (*Acheta domesticus*). Octanoic acid is the main toxic compound of the fruit of *Morinda citrifolia*, where it functions as a lethal or toxic defense against most drosophilids, with the exception of *Drosophila sechellia*, which has evolved physiological adaptations allowing it to specialize on the “toxic” Morinda fruit. Octanoic acid has also been found to act as negative signal for ovipositional responses of female mosquitoes [17,18] and as a repellent for the turnip moth *Agrotis segetum* [19].

The acid is also accumulated by yeasts as a by-product of ethanolic fermentation, and the concentrations produced, although low (<20 mg/L), are highly toxic for the yeast itself [20,21]. However, no research data currently exist about the production of octanoic acid by *C. coronatus*.

The greater wax moth, *G. mellonella*, is well known for its parasitization of honeybees and the consequent economic loss [22], and there is a need for new, safe, and nontoxic methods of controlling it. Octanoic acid seems to be a suitable candidate for this role, as it is harmless and non-hazardous to bees [23], and is not dangerous for humans, as indicated by its role as a food additive. Therefore, the present study examines the insecticidal activity of octanoic acid against *G. mellonella*.

*G. mellonella* is an interesting research object, not only because of its role as a pest but also because it is considered as a surrogate model for the investigation of toxicity of toxins or food additives [24,25]. Due to the similarities between invertebrate and mammalian innate immune systems, it has also been utilized as a model system for studying the virulence of microbial pathogens and for assessing the efficacy of anti-microbial drugs; indeed, it has been found to yield results comparable to those in mammal models [26,27,28,29,30,31]. Despite this, no research data exist regarding the use of *G. mellonella* as an alternative model to examine the effect of octanoic acid. As such, there is a great need to conduct such studies.

Pathogens interfere with a wide range of signalling pathways to overcome host cell-mediated defense mechanisms such as cell death [32,33,34,35,36,37,38,39]. The immunocompetent cells, hemocytes, become necrotic or undergo apoptosis as immune defense responses to foreign organisms and toxic compounds [40,41,42]. Apoptosis is involved in a range of cellular processes; however, these fall into two broad categories: the development and shaping of the immune receptor repertoire and immune effector mechanisms. Although its effects on the immune system have been primarily observed during viral infections [43], they are also believed to play a key role during fungal infection [44].

Studies have found that insect species, such as *D. melanogaster* and *G. mellonella*, are able to produce reactive oxygen species (ROS) [45,46,47]. These molecules have been employed in insect defense systems as cytotoxic agents against pathogens [48,49,50,51,52] or insecticide [53]. In addition, the presence of homologous components between insect hemocytes and human neutrophils and their effects on ROS generation processes have been reported [54,55,56].

*G. mellonella* ROS production occurs mainly in hemocytes, which are responsible for phagocytosis and pathogen destruction through the production of superoxide radicals, nitric oxide, and hydrogen peroxide [46,54,57]. However, ROS can also be found free in *G. mellonella* hemolymph, because they are produced during melanization; more precisely, they are generated by enzymatic oxidation–reduction reactions resulting from the activation of the phenoloxidase cascade, which produces hydrogen peroxide and superoxide [45,58,59].

The main aim of the present work was to examine the impact of octanoic acid, another identified metabolite of C. coronatus, on *G. mellonella* larvae and adults. The testable hypothesis was that exposure to octanoic acid might affect the two major antifungal defense systems of insects: cuticular FFA profiles and hemocytes, both being crucial elements of immune system.

## 2. Results

### 2.1. The Insecticidal Activity of Fungal Filtrates against G. mellonella Larvae and Adults

**Figure 1:**
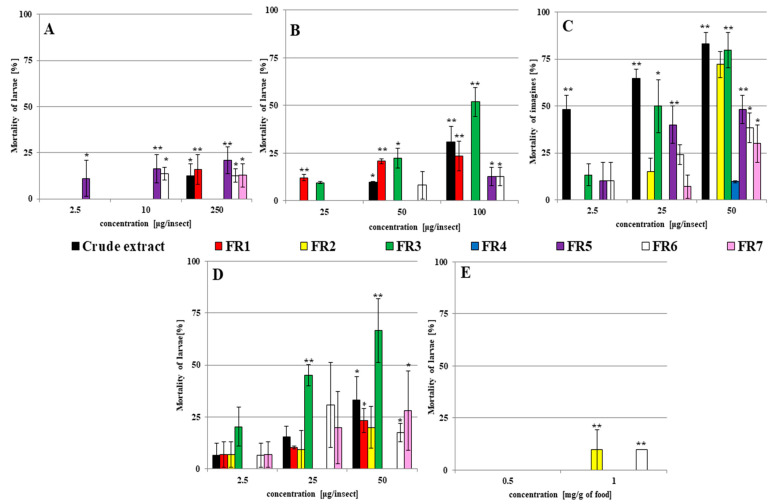
**The insecticidal activity of fractions extracted from *C. coronatus* post-incubation filtrate against *G. mellonella*.** (**A**) Topical application of fractions dissolved in acetone to larvae. (**B**) Topical application of fractions dissolved in ethanol to larvae. (**C**) Topical application of fractions dissolved in ethanol to adults. (**D**) Injection of fractions dissolved in DMSO to larvae. (**E**) Application of fractions dissolved in ethanol to food of larvae; FR1-Fraction 1; FR2-Fraction 2; FR3-Fraction 3; FR4-Fraction 4; FR5-Fraction 5; FR6-Fraction 6; FR7-Fraction 7; statistically significant differences (ANOVA, Dunnett test) between the mortality of *G. mellonella* larvae (**A**,**B**,**D**,**E**) and adults (**C**) after topical application (**A**–**C**), injection (**D**), and addition to the food (**E**) and controls (1 µL of acetone or ethanol to topical applications, 1 µL of DMSO to injection, and 5 mL of ethanol to 4 g of food samples) are marked * for *p* < 0.05 and ** for *p* < 0.001; raw data are provided in Table S1.

**Figure 2:**
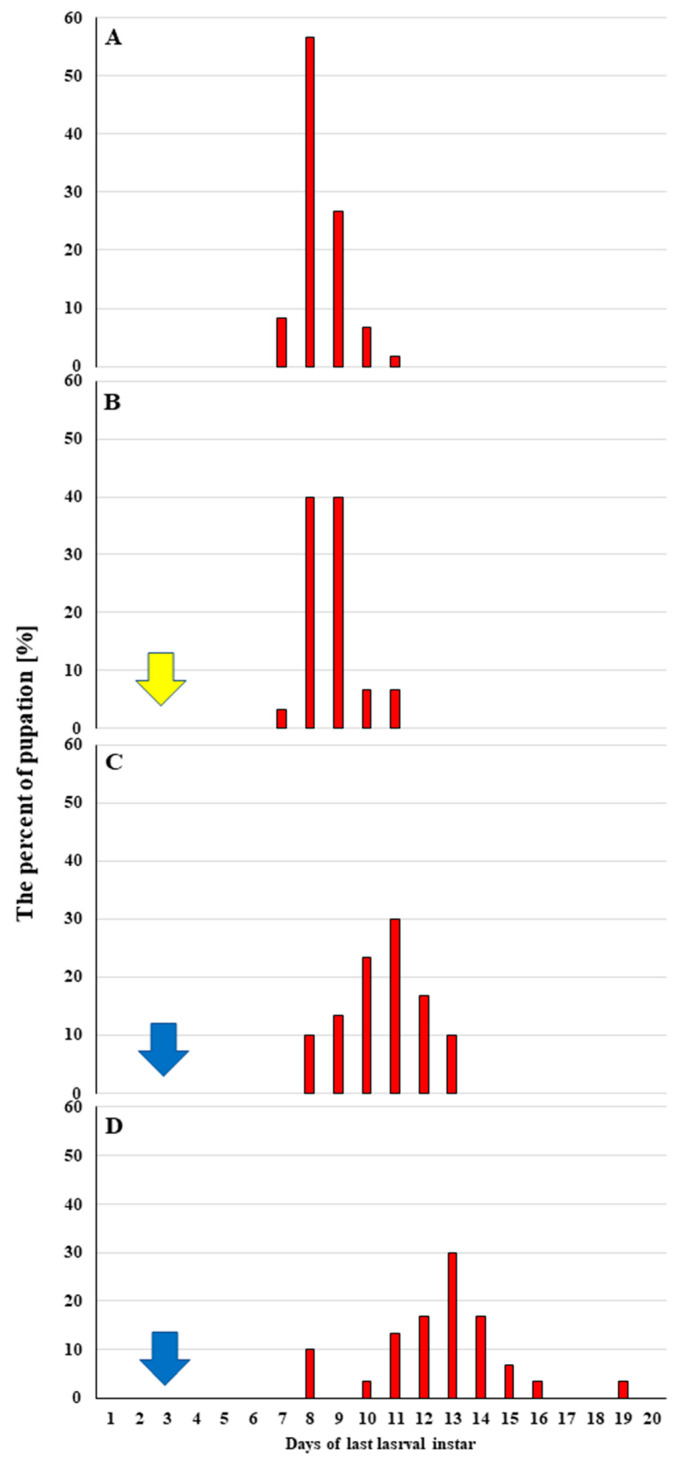
**Pupation of *G. mellonella* last instar larvae fed food contaminated with *C. coronatus* FR3 extract.** (**A**) untreated larvae, (**B**) food contaminated with ethanol, (**C**) food contaminated with FR3 (0.5 mg/g of food), (**D**) food contaminated with FR3 (1 mg/g of food). FR3-Fraction3. Arrows indicate the day of administration of the contaminated food: blue arrow FR3, yellow arrow ethanol.

During the extraction of *C. coronatus* filtrate, seven fractions were obtained. Their insecticidal activity was checked against *G. mellonella* larvae and adults; the results are presented in Figure 1 and Figure 2, and raw data are shown in Table S1.

Two types of solvent were used: acetone and ethanol. Preliminary studies have shown that neither solvent affects the mortality of larvae; therefore, the insecticidal activity of the crude extract and each fraction against larvae was checked after dissolution in acetone (Figure 1A) and ethanol (Figure 1B). The insecticidal activity of individual fractions was higher when ethanol was used as a solvent. The most active fraction was FR3: the application of 100 µg per insect (with ethanol as a solvent) resulted in 51.82 ± 7.44% mortality among the larvae compared to controls (*p* < 0.001).

Acetone was toxic for *G. mellonella* adults and caused the death of 28.33 ± 7.52% insects (Supplementary Table S1); therefore, for topical application of fractions to adults, ethanol was chosen as a solvent. The results are shown in Figure 1C. The examined fractions were more toxic for adults than for larvae. The highest insecticidal activity was observed for crude extract, FR2, and FR3 (all 50 µg/insects), which caused the death of 83.33 ± 5.77%, 72.22 ± 6.94, and 79.63 ± 9.45% of the examined adults, respectively (in all *p* < 0.001).

Fraction FR3 had also the highest insecticidal activity, resulting in 20.37 ± 9.45, 45.15 ± 5.01, and 66.67 ± 15.28% mortality (for all *p* < 0.001) after injections of 2.5, 25, and 50 µg/insect, respectively (Figure 1D).

When the fractions were administered in contaminated food, mortality was only observed for FR2 and FR6 at 1 mg/g of food (Figure 1E). While no lethal effect was observed for FR3, its application at concentrations of 0.5 and 1 mg/g of food resulted in pupation being delayed by 2.67 ± 1.35 days and the last larval instar being extended by 4.73 ± 2.13 days compared to the controls (Figure 2). These results were not observed after treatment with other fractions.

Hence, FR3 demonstrated the highest insecticidal activity: Topical applications and injection of this fraction caused the highest mortality among larvae and adults, and food intake disturbed metamorphosis by extending the time needed for pupation. This fraction was chosen for further analysis.

### 2.2. The Detection of Octanoic Acid in FR3

**Figure 3:**
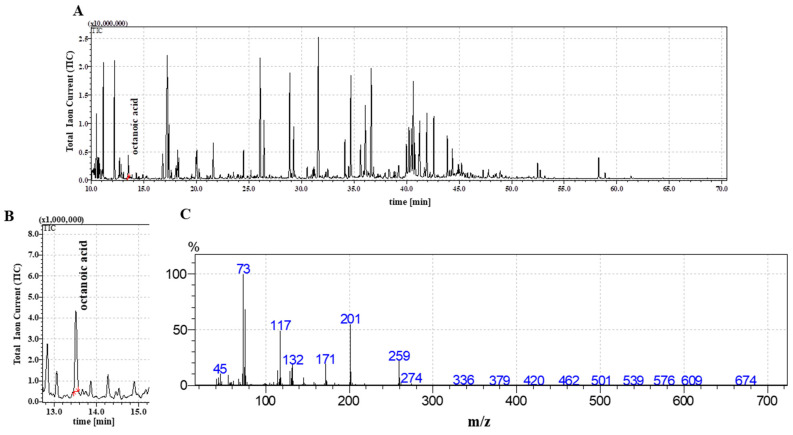
**GC/MS analysis of FR3.** (**A**) The total ion current (TIC) chromatogram of trimethylsilyl esters (TMS esters) of the extract from fraction 3 (FR3) *C. coronatus* filtrate; (**B**) The localization of the trimethylsilyl ester of octanoic acid on the chromatogram; (**C**) The mass spectrum of the trimethylsilyl ester of octanoic acid, C8:0.

The FR3 fraction extracted from *C. coronatus* filtrate was analyzed by GC-MS, as described in Materials and Methods; the results are provided in Figure 3A. The mass spectra of the trimethylsilyl esters of C8:0 comprised M + (molecular ion), m/z = 216, [M-15] +, m/z = 201, and fragment ions at m/z 117, 129, 132, and 145 and retention time 13.45 min; the results confirmed the presence of C8:0 (Figure 3B,C).

### 2.3. The Insecticidal Activity of Octanoic Acid

#### 2.3.1. Effect of Octanoic Acid on the Survival and Further Development of *G. mellonella* Larvae

*G. mellonella* larvae were given C8:0 by topical application in two doses (50 and 100 μg per one larva), and the survival rates and metamorphosis dynamics of surviving individuals were calculated (Table 1). The raw data are provided in Table S2. No statistically significant difference was found between untreated controls (C1) and solvent-administered controls (C2; acetone used as a solvent). Day “0” indicates the first day of the last larval instar.

The topical application of octanoic acid had a dose-dependent influence on *G. mellonella* survival. The administration of 50 µg per insect did not result in any effect on survival level: 93.33 ± 6.24% survival among the treated insects, 95.33 ± 0.47% for untreated control (C1), and 95.67 ± 3.30% for acetone control (C2); however, a double dose (100 µg per insect) decreased the percentage of surviving larvae by 3.3-times (28.33 ± 22.48%, F(3, 8) = 15.909, *p* = 0.00098). The results are provided in Table 1.

The topical administration of 100 µg octanoic acid affected also the pupation and the emergence of *G. mellonella* adults (Table 1). Significantly lower percentages of pupation (20.00 ± 18.71%, F(3, 8) = 15.995, *p* = 0.00097,) and adult eclosions (11.67 ± 10.27%, *p* < 0.05) were observed compared to both controls. Increasing the dose to 100 µg reduced the time required for pupation and adult eclosion; however, no significant differences in mean pupation time or time to emergence were observed between the control group and insects treated with C8:0 (Table 1).

#### 2.3.2. The LD50 and LD100 Values of Octanoic Acid for *G. mellonella* Larvae and Adults

**Figure 4:**
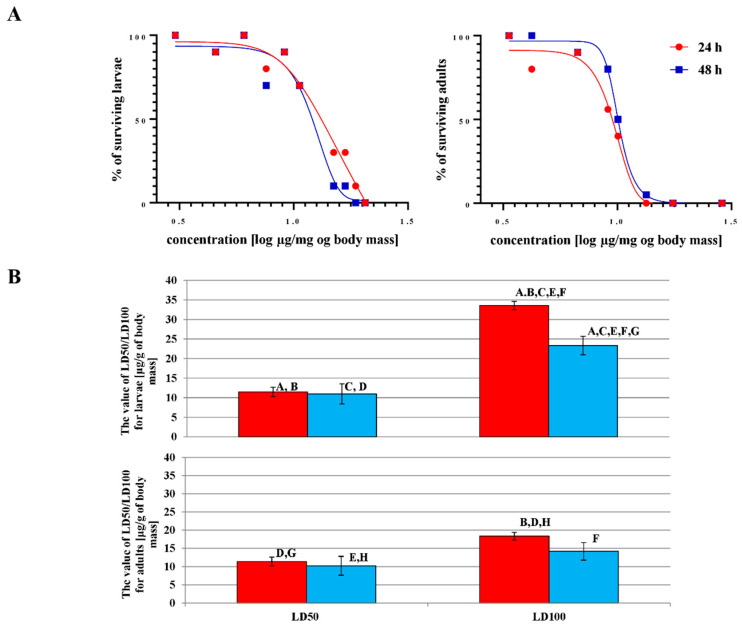
**Assessment of the toxicity of octanoic acid to *G. mellonella* larvae and adults.** (**A**) Dose–response surviving curve of topical application of octanoic acid to larvae and adults. The percentage of surviving insect values is plotted against the log of concentration of the compounds. The curves for the surviving levels of insects were fitted to a non-linear log-logistic regression model using GraphPad Prism,v. 6.0 (GraphPad Software); (**B**) the value of LD50/LD100 parameter for larvae and adults after treatments with octanoic aicd; LD50—lethal dose 50; LD100—lethal dose 100; Statistically significant differences between LD50 and LD100 doses after octanoic acid topical application (ANOVA, Test HSD Tukey, *p* < 0.05) are marked with the same letters (A,B,C,D,E,F,G,H); the raw data are provided in Table S3.

Two parameters (LD50 and LD100) were calculated to determine the toxic effect of octanoic acid on *G. mellonella* larvae and adults. The results are presented in Figure 4 and in Table S3.

Octanoic acid was more toxic to adults than larvae. A lower LD100 value (14.18 ± 1.72 µg/mg of body mass) was observed for adults (Figure 4B), and the effect was time-dependent, i.e., the LD100 value fell as incubation time increased. Significantly higher LD100 parameters were observed after topical administration of octanoic acid to larvae (Figure 4A) than in adults. However, statistically significant differences in LD50 were found between larvae and adults or between 24 and 48 h of incubation in either development stages.

### 2.4. The Changes in Cuticular FFA Profiles Observed in G. mellonella after Topical Application of Octanoic Acid (LD50 or LD100 Dose)

The concentrations of the individual FFAs extracted from larvae and adults are presented in Table 2 (raw data in Table S4).

In the larvae, the highest total FFA content was observed after treatment with octanoic acid at LD50 (1820.25 ± 83.96 μg/g of insect body); this value was 6.01 times higher than in the untreated control C1 (299.10 ± 18.03 μg/g of insect body), 16.46 times higher than in acetone treated control C2 (110.61 ± 13.37 μg/g of insect body), and 2.44 times higher than after treatment with LD100 (745.11 ± 331.50 μg/g of insect body; F(3,8) = 39.97, df = 8.00, *p* < 0.001). In contrast, in adults, the highest cuticular total FFA content was observed in the C1 control (277.04 ± 3.51 μg/g of insect body); the value was 1.63 times higher than in the extract from insects treated with ethanol (169.65 ± 16.23 μg/g of insect body), 1.53 times higher than after octanoic acid treatment with the LD50 dose (181.26 ± 32.13 μg/g of insect body), and 1.1 times higher than in insects treated with LD100 (251.80 ± 40.66 μg/g of insect body) (F(3,8) = 5.05, df = 8.00, *p* = 0.03).

FFAs C16:0, C18:2, and C18:1 were found to be dominant in the cuticular extracts from untreated larvae and adults. C14:0, C16:0, C18:2, and C18:1 predominated in extracts from larvae treated with acetone or octanoic acid, while C11:0, C14:0, C16:1, C16:0, C18:1, and C18:2 predominated in extracts from adults treated with ethanol or octanoic acid.

The cuticular extracts from the untreated larvae (C1) contained 17 FFAs from C6:0 to C18:0: ten saturated (C6:0, C8:0–C10:0, C12:0, C14:0–C18:0) and seven unsaturated (C10:1, C14:1, C16:1, C17:1, C18:3, C18:2, and C18:1). Four FFAs (C10:1, C17:1, C18:3, and C18:0) were detected in the extract from untreated control larvae.

The cuticular extracts from the untreated control adults contained twenty-two FFAs; eight of these were not found in treated adults and disappeared after treatment (C10:1, C14:1, C17:0, C18:3, C20:1, C20:0, C24:0, and C26:0). Some differences were observed between untreated larvae and adults: for example, C11:0, C13:0, C20:1, C20:0, C24:0, and C26:0 were present in the control adult extract but not in control larvae, while C17:1 was present in adults but not larvae.

Twelve FFAs were detected in C2 control larvae; however, acetone treatment resulted in the loss of C10:1, C17:1, C18:3, C18:2, and C18:0, as well as a significant elevation of the four cuticular FFAs C14:1, C14:0, C15:0, and C16:0; among the latter, the increase ranged from a 2.4-fold (C14:1) to a 13-fold (C15:0) increase.

After ethanol treatment, 13 FFAs were detected in the adults; of these, C10:1, C14:1, C17:0, C18:3, C18:2, C18:0. C20:1, C20:0, C24:0, and C26:0 were absent from the C1 control group. Contact with ethanol resulted in a significant increase in the concentrations of seven cuticular FFAs: C10:0 (6.99-fold increase), C11:0 (2.04-fold increase), C12:0 (increase) C14:0 (increase), C9:0 (1.43-fold decrease), C18:1 (4.40-fold decrease), and C18:0 (4.28-fold decrease). An increase in C14:0 was also observed in larvae after acetone treatment.

The lipid extracts obtained from larvae after octanoic acid treatment were found to contain 14 FFAs (LD50) and 10 FFAs (LD100; Table 2). FFAs C14:1 and C17:0, which were present in both control fractions, were absent after octanoic acid treatment. In addition, C11:0 and C13:0 were detected only after the application of the LD50 dose. Significant elevations in FFA C8:0, C10:0, C12:0, C14:0, C16:1, and C18:2 levels were observed after octanoic acid treatment.

The lipid extract obtained from adults after octanoic acid treatment was found to contain 14 FFAs after treatment with either dose: LD50 and LD100 (Table 2). Significant increases in C8:0, C15:0, and C16:1 were observed, and significant decreases were observed in C6:0, C9:0, and C18:2. Of these, 15:0 and C16:1 were also found to be elevated in larvae treated with octanoic acid.

### 2.5. Cytotoxic Effect of Octanoic Acid on Sf-9 Cell Line

Preliminary studies on the effect of octanoic acid on the survival of *G. mellonella* hemocytes in vitro demonstrated that commercially available tests were unsuitable for use in these cells: the hemocytes failed to proliferate in vitro, and the cultures themselves were highly heterogeneous, being composed of five hemocyte classes. For this reason, the stable insect cell line Sf-9 was chosen to determine the cytotoxic effects of octanoic acid.

**Figure 5:**
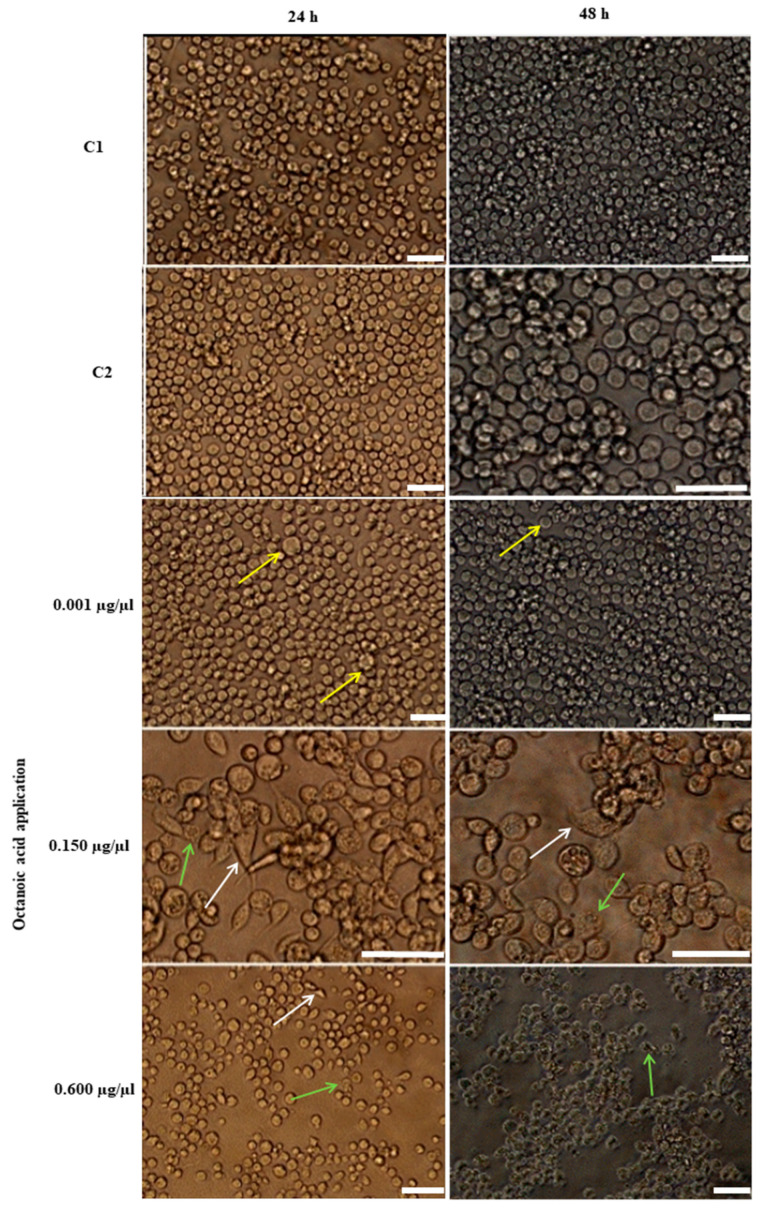
**The changes in Sf-9 cell morphology after treatment with octanoic acid.** C1—untreated cells; C2—cells treated with ethanol; scale bar 100 μm; arrows indicate the changes in morphology of Sf-9 cells: white arrows indicate spindle-shaped cells, green arrows indicate cell degranulation, and yellow arrows indicate cells with higher volume, scale bar 25 µm.

Changes in the morphology of Sf-9 cells after in vitro administration of octanoic acid and after 24 or 48 h of incubation are presented in Figure 5. After application, the cells were observed to change shape to elongated and spindle-shaped. Moreover, an increase in cell volume and/or degranulation was detected after treatment.

The cytotoxicity of the tested compounds against the Sf-9 insect cell line was indicated by LC50, i.e., the concentration of the toxic substance causing the death of half of the tested population. In this case, the value represents the concentrations of octanoic acid causing 50% of cell death in the culture. Cell viability was assessed by WST-1 (indicating cell metabolic activity) and LDH tests (indicating cell membrane continuity).

**Figure 6:**
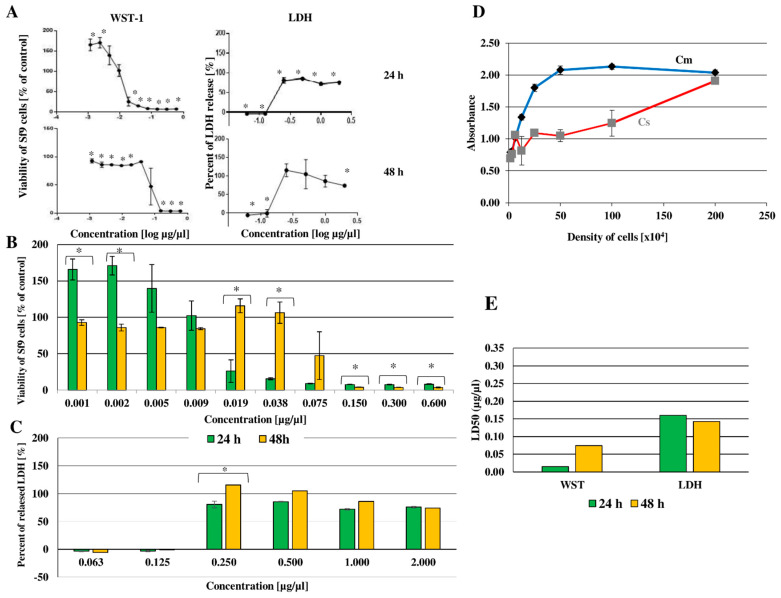
**Cytotoxic effect of octanoic acid on the Sf-9 cell line.** (**A**) Changes in viability of Sf-9 cells after treatment with various concentrations of octanoic acid as determined using the WST-1 and LDH tests. Statistically significant differences in viability between cells treated with octanoic acid and C1 controls (Student’s *t*-test, *p* < 0.05) are marked with *. The curves for the effect on Sf-9 cells were fitted to a non-linear log-logistic regression model by using GraphPad Prism, v. 6.0 (GraphPad Software); (**B**) changes in the viability of Sf-9 cells after 24 and 48 h of incubation with various concentrations of octanoic acid (measured by the WST-1 test). Statistically significant differences between the viability of Sf-9 cells after 24 h and 48 h of incubation (Student’s *t*-test, *p* < 0.05) are marked with *. (**C**) Changes in LDH released from Sf-9 cells after 24 and 48 h of incubation with various concentrations of octanoic acid. Statistically significant differences between the percent of LDH released from Sf-9 cells after 24 h and 48 h of incubation (Student’s *t*-test, *p* < 0.05) are marked with *. (**D**) The determination of optimal Sf-9 cell density for the LDH test. Cm—maximum LDH release; Cs—spontaneous enzyme release. (**E**) The lethal dose (LC50) of octanoic acid after 24 and 48 h of incubation with Sf-9 cells, measured with WST-1 and LDH tests.

The WST-1 test was used to determine cell viability after octanoic acid treatment 24 and 48 h after administration; the percentage of viable cells is given against the log of the concentration of the tested compound in Figure 6A (raw data in Table S5).

After the application of octanoic acid, a significant change in cell viability was detected after 24 and 48 h of incubation compared to control cells. The results are shown in Figure 6B (raw data in Table S5); longer incubation times were associated with lower cell death rates after using the three highest concentrations (0.600, 0.300, and 0.150 μg/μL; for all *p* ≤ 0.001) and two lowest (0.001 and 0.002 μg/μL, *p* < 0.001). However, the opposite situation was observed at concentrations of 0.075, 0.038 (*p* < 0.001), or 0.019 μg/μL (*p* = 0.002); in this case, longer incubation was associated with greater cell viability.

For the LDH test, the initial stage of the experiment was to select the optimal cell density. The relationship between absorbance and Sf-9 cell concentration is presented in Figure 6D. Two controls were used: Cm, i.e., cells treated with lysing enzyme, indicating maximum lactate dehydrogenase secretion, and Cs, i.e., untreated cells, indicating spontaneous secretion. The optimal density was selected as 5.0 × 10^5^ cells.

Changes in LDH level following the in vitro administration of octanoic acid to Sf-9 cells are given in Figure 6A (raw data in Table S5). The results are presented as the relationship between the log of octanoic concentration and the percentage of enzyme released. The maximum release of lactate dehydrogenase, indicated by Cm, was taken as 100%. Octanoic acid administration was found to influence LDH release: this value decreased for octanoic acid concentrations 0.06 and 0.13 μg/μL (*p* < 0.001) but increased (from 73.64 to 83.49% of Cm) at concentrations of 25 to 2.00 μg/μL (*p* < 0.001).

The effect of incubation time on the percentage released LDH from Sf-9 cells is shown in Figure 6C, indicating the relationship between the concentration of the test compound and the percentage of released LDH. The absorbance for the cells treated with the lysing enzyme was assumed as 100%, i.e., the control of the maximum release of the tested enzyme. A score below 0 on the *x*-axis indicates that the percentage of LDH released in the tested samples was lower than in the control for spontaneous release (i.e., Cs—untreated Sf-9 cells). After the use of octanoic acid, an increase in LDH concentration in the medium was observed at a concentration of 0.25 μg/μL both after 24 and 48 h of incubation (*p* = 0.03).

#### Lethal Concentration (LC50)

Based on the viability test results, LC50 was determined with GraphPad (Prism). The results are presented in Figure 6E, showing the LC50 value against Sf-9 cells after 24- and 48-h incubation with octanoic acid. Large discrepancies in LC50 were observed between the applied WST-1 test and the LDH for both incubation times, with the test determining cell metabolic activity of cells yielding much lower than for the test determining the permeability of cell membranes.

After the administration of octanoic acid, LC50 was found to change depending on the incubation time. For the WST-1 test, cytotoxicity was found to increase sevenfold for the longer incubation time: increasing from 0.01 μg/μL after 24 h to 0.07 μg/μL after 48 h. For the LDH assay, LC50 fell slightly from 0.16 µg/ µL at 24 h to 0.15 μg/μL after 48 h.

### 2.6. The Changes in G. mellonella Hemocytes Observed after Octanoic Acid Application

#### 2.6.1. Morphological Changes in Hemocytes after Octanoic Acid Application

**Figure 7:**
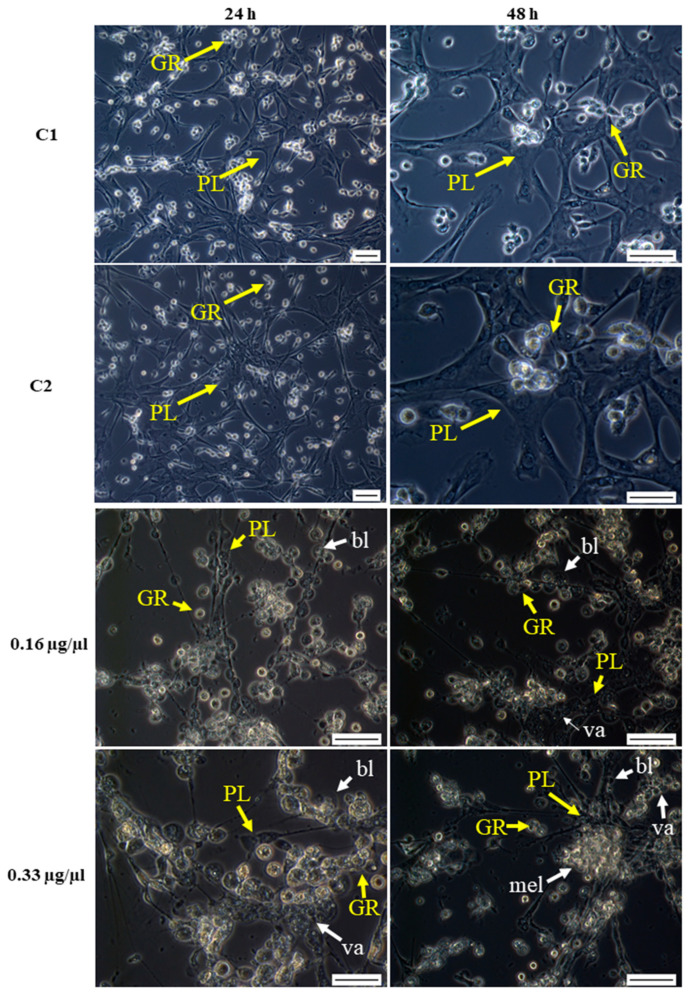
**The morphological changes in *G. mellonella* hemocytes cultured with octanoic acid in vitro.** C1—untreated cells, C2—ethanol control; PL—plasmatocytes; GR—granulocytes; bl—cells blebs; mel—melanization; va—vacuolization of cells, scale bar 25 μm.

Octanoic acid was given directly to cell cultures at final concentrations of 0.16 or 0.33 µg/ µL: These concentrations were closest to those used for the assays on the Sf-9 cell line. Morphological changes were observed after 24 and 48 h of incubation and are shown in Figure 7. Mostly, the tested compound appeared to have a destructive effect on plasmatocytes and granulocytes resulting in the destruction of the hemocyte nework, the loss of their cellular skeleton, and the creation of cell aggregates. In addition, granulocyte degranulation and disintegration was observed (Figure 7).

**Figure 8:**
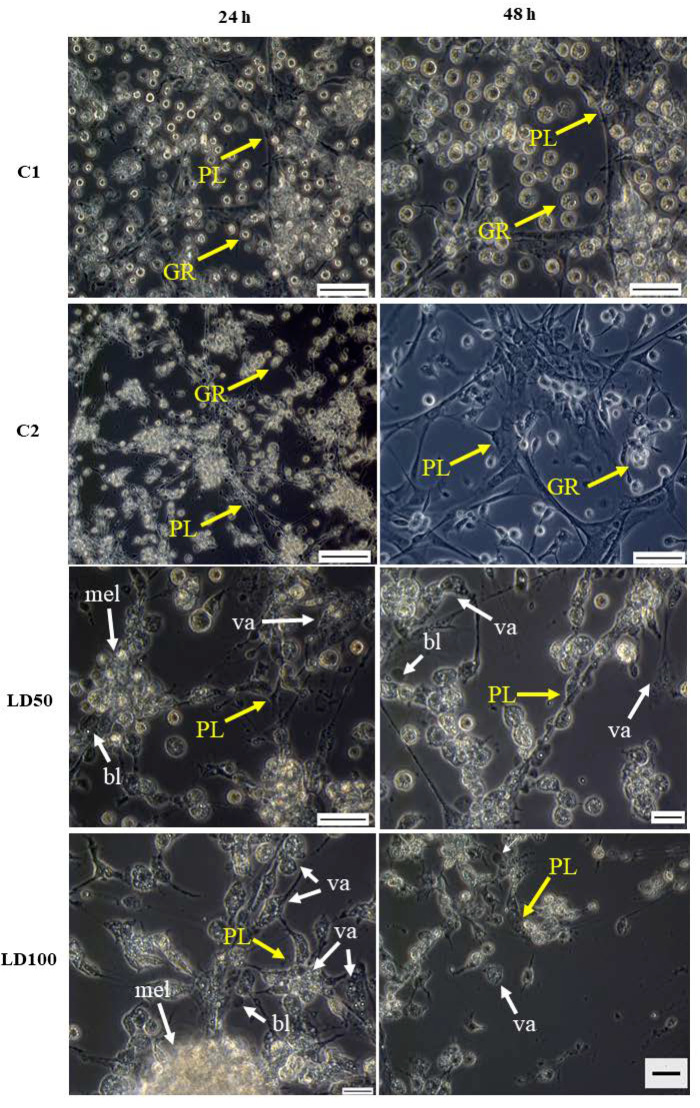
**The morphological changes in *G. mellonella* hemocytes taken from larvae treated with octanoic acid.** C1—untreated cells; C2—acetone control; PL—plasmatocytes; GR—granulocytes; bl—cells blebs; mel—melanization; va—vacuolization of cells, scale bar 25 μm.

The administration of octanoic acid to *G. mellonella* larvae also had a destructive influence on hemocyte morphology (Figure 8). Octanoic acid administration inhibited networking and significantly delayed network formation: The hemocytes were still visible 24 h later than in the control. The tested compound changed all classes of hemocytes, causing their vacuolization, cytoplasmic ejection, and consequently cell lysis (Figure 8).

#### 2.6.2. Detection of Apoptosis in *G. mellonella* Hemocytes after Octanoic Acid Application

Apoptosis was detected in *G. mellonella* hemocytes after octanoic acid application (both in vivo and in vitro) using Annexin V, a protein that selectively recognizes phosphatidylserine (PS). Under physiological conditions, this phospholipid is present in the cell membrane on the cytosolic side; however, it is transferred to the outside of the cell membrane during the early phase of apoptosis. Annexin V was conjugated with the green fluorescent protein (GFP), which allows for its precise detection in a fluorescence microscope. Necrosis was detected using 7-aminoactinomycin D (7-AAD): The compound penetrates through the loosened membrane inside the cell and stains DNA. The presence of 7-AAD in the nucleus is an indicator of late apoptosis or necrotic cells. Additionally, the cell nuclei were stained with Hoechst dye.

**Figure 9:**
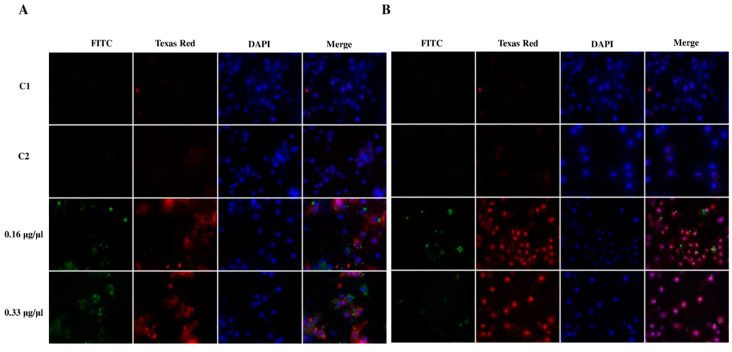
**The detection of apoptosis/necrosis in *G. mellonella* hemocytes after in vitro administration of octanoic acid.** (**A**) After 24 h of incubation; (**B**) after 48 h of incubation with the tested compound; C1—untreated cells; C2—ethanol control; apoptotic cells (green) were stained by Annexin V; cell nuclei (blue) were stained with Hoechst dye; necrotic cells (red) were detected using 7-aminoactinomycin D (all as a part of GFP CERTIFIED^®^Apoptosis/Necrosis Detection Kit by Enzo Life Sciences), scale bar 25 μm.

Apoptosis and/or necrosis were observed 24 and 48 h from the direct addition of octanoic acid to *G. mellonella* hemocyte cultures. The octanoic acid was added at a final concentration of 0.16 or 0.33 μg/μL, i.e., the same values used to evaluate the morphological changes after application. The changes taking place after 24 h are presented in Figure 9A. Green fluorescence was observed on the FITC channel and low-intensity red fluorescence on the Texas Red channel after both concentrations of acid, indicating the presence of apoptotic cells. After 48 h (Figure 9B) of incubation, a higher intensity of red fluorescence was observed, mostly in the cell nuclei, indicating the occurrence of necrosis or late apoptosis.

**Figure 10:**
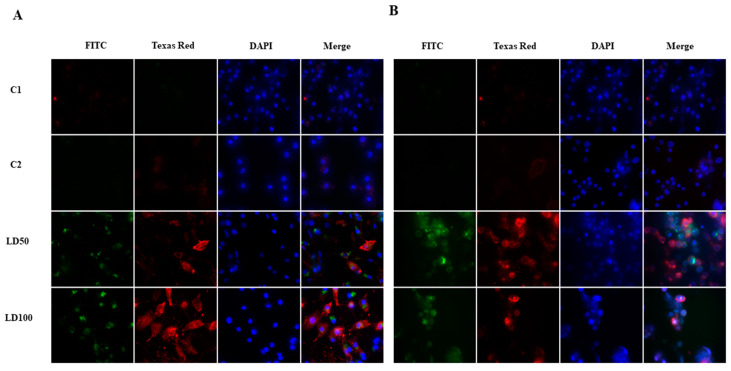
**The detection of apoptosis/necrosis in *G. mellonella* hemocytes taken from octanoic acid-treated larvae.** (**A**) After 24 h of incubation; (**B**) after 48 h; C1—untreated insects; C2—acetone control. Apoptotic cells (green) were stained by Annexin V; cell nuclei (blue) were stained with Hoechst dyes; necrotic cells (red) were detected using 7-aminoactinomycin D (all as a part of GFP CERTIFIED^®^Apoptosis/Necrosis Detection Kit by Enzo Life Sciences), scale bar 25 μm.

The administration of LD50 and LD100 octanoic acid to the *G. mellonella* larvae also induced the occurrence of apoptosis/necrosis in hemocytes (Figure 10). After 24 h of *G. mellonella* cell culture, green (FITC channel) and red (Texas Red channel) fluorescence were observed (Figure 10A), suggesting the occurrence of apoptosis and/or necrosis; this was particularly apparent after the LD100 dose. Increasing the time of incubation to 48 h resulted in higher intensity red fluorescence in the Texas Red channel compared to 24 h; however, 7-AAD was not bound to the cell nuclei (Figure 10B).

#### 2.6.3. Identification and Measurement of Caspase Activity in *G. mellonella* Hemocyte Cultures after Octanoic Acid Application

Caspase activity was detected using FAM-VAD-FMK, a carboxyfluorescein derivative of valylalanylaspartic acid fluoromethyl ketone (VAD-FMK), which is a potent inhibitor of caspase activity. The VAD multi-caspase substrate is used to detect caspases 1–9. After entering the cell, the FAM-VAD-FMK reagent forms a covalent bond with Cys 285 residue on the large subunit of the caspase heterodimer. Following this, the FAM caspase inhibitor binds to any active caspase enzymes in the cell; however, during the labeling process, the inhibitors remain cell-permeable and noncytotoxic. In apoptotic cells, the probes attach a green fluorescent label to the active site of the caspase enzyme; they can now be detected under a fluorescence microscope and by 96-well fluorometry.

**Figure 11:**
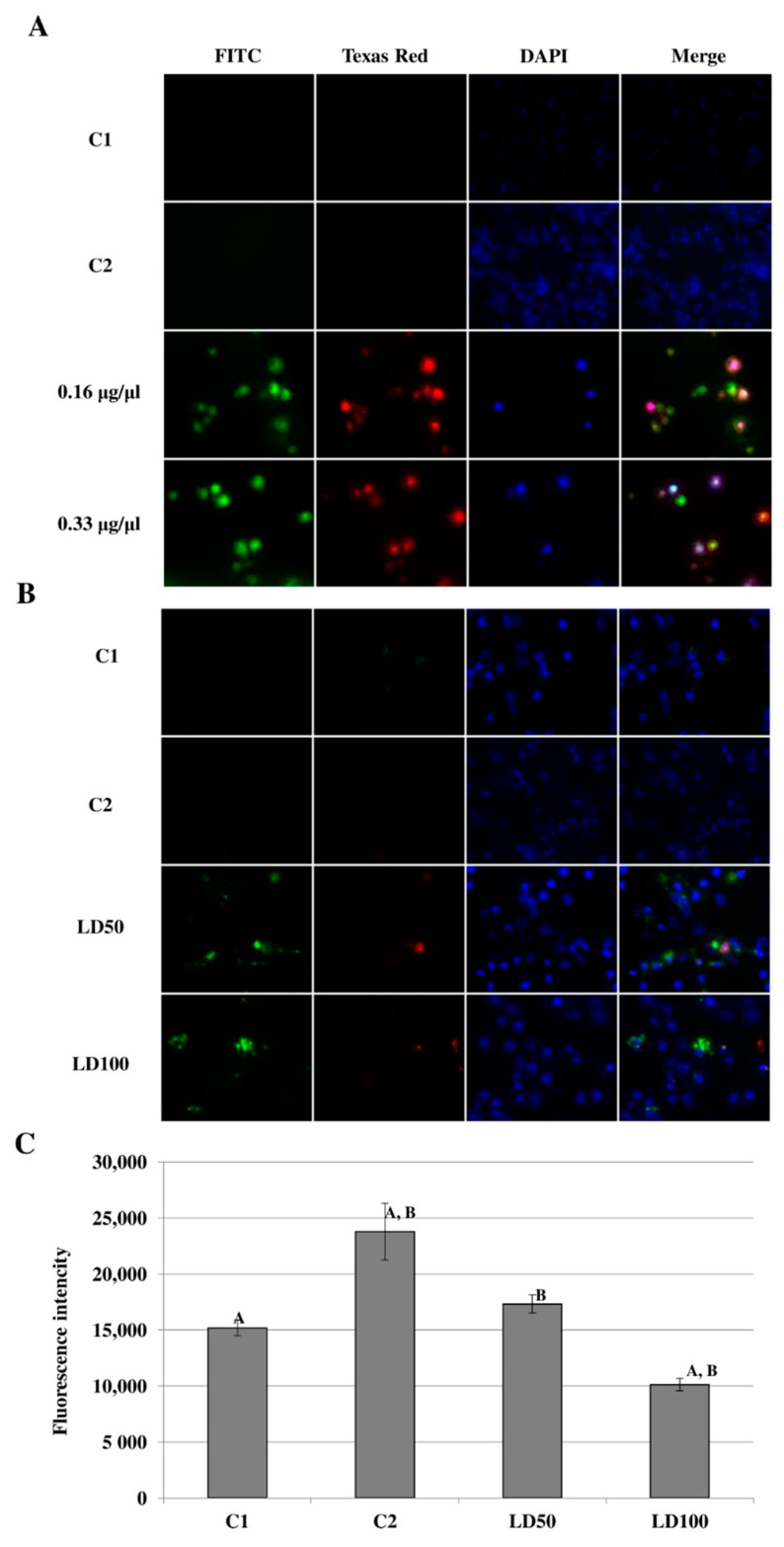
**The detection and measurement of caspase activity in *G. mellonella* hemocytes after octanoic acid application.** (**A**) Detection of caspase activity after in vitro administration of octanoic acid; (**B**) detection of caspase activity in hemolymph from octanoic acid-treated larvae (in vivo application of octanoic acid); (**C**) measurement of caspase activity in hemolymph from octanoic acid-treated larvae (in vivo application of octanoic acid); C1—untreated cells, C2—acetone (in vivo)/ethanol (in vitro) control; active caspases (green) were stained by carboxyfluorescein derivative of valylalanylaspartic acid fluoromethyl ketone (VAD-FMK). Cell nuclei (blue) were stained with Hoechst dye. Necrotic cells (red) were detected using propidium iodide (all as a part of Carboxyfluorescein MultiCaspase Activity Kit by Enzo Life Sciences), scale bar 25 μm; statistically significant differences are marked with the same letters (A,B) (ANOVA, Test HSD Tukey, *p* < 0.05); raw data are provided in Table S6.

The in vitro addition of octanoic acid directly to the *G. mellonella* hemocyte cultures resulted in caspase activation (Figure 11A). The activity of the caspases was compared at two octanoic acid concentrations: 0.16 and 0.33 μg/μL (final concentration in the culture). After the addition of octanoic acid, green fluorescence was observed in the FITC channel with the intensity being dependent on the dose of the test compound. Red staining of cell nuclei was visible in the image from the Texas Red channel, suggesting that necrosis or late apoptosis is taking place in these cells.

The administration of octanoic acid also influences the activation of caspases in vivo (Figure 11B). The insects were given octanoic acid in two concentrations corresponding to the LD50 and LD100 doses, after which, green fluorescence was observed on the FITC channel, indicating the presence of active cysteine proteases in the culture. Weak red fluorescence was also observed on the Texas Red channel.

Spectrofluorometric testing also revealed a significant increase in caspase activity (Figure 11C; raw data in Table S6; ANOVA, Test HSD Tukey, F(3, 8) = 32.71, *p* < 0.001) after the administration of solvent (acetone) to larvae from 15,207.33 ± 685.56 for C1 to 23,801.67 ± 2537.29 for C2 (*p* = 0.001). Although the administration of the LD50 dose did not result in any significant difference in fluorescence intensity (17,338.00 ± 798.75, *p* = 0.47), the higher dose (LD100) was associated with a decrease in caspase activity (10,117.67 ± 557.43, *p* = 0.03).

#### 2.6.4. The DNA Damage Observed in *G. mellonella* Hemocytes after In Vivo Application of Octanoic Acid to Larvae

**Figure 12:**
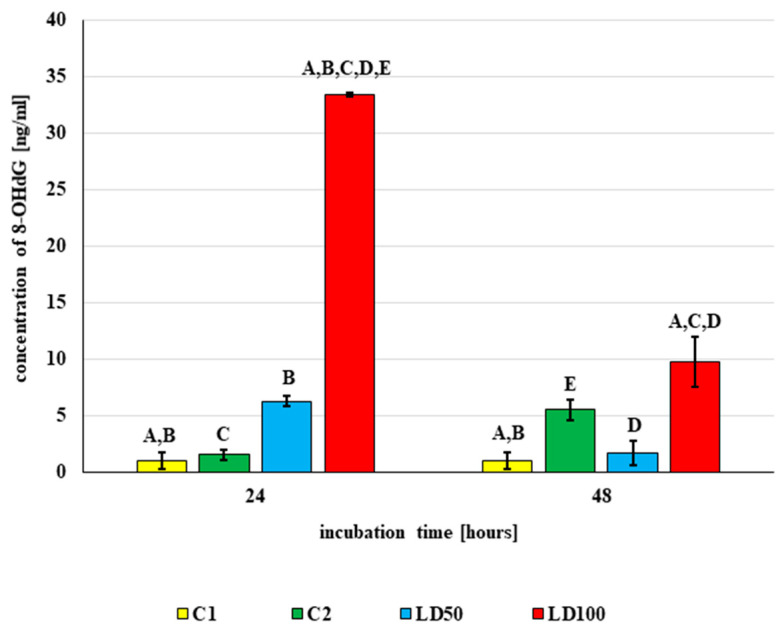
The changes in 8-hydroxy-2’-deoxyguanosine (8-OHdG) concentration in *G. mellonella* hemocytes after octanoic acid application. C1—untreated cells; C2—acetone control; statistically significant differences are marked with the same letters (A,B,C,D,E) (ANOVA, Tukey’s HSD Test, *p* < 0.05); raw data are given in Table S7.

The modified nucleoside base 8-hydroxy-2′-deoxyguanosine (8-OHdG) is used as a biomarker of oxidative stress. In vivo application of octanoic acid changed the concentration of 8-OHdG in *G. mellonella* hemolymph (ANOVA, Test HSD Tukey, F(6, 10) = 129.00, *p* = 0.000), as noted in Figure 12. In addition, no statistically significant differences in 8-OHdG concentration were observed between untreated larvae (C1—0.96 ± 0.74 ng/mL) and those treated with acetone (C2): i.e., 1.55 ± 0.43 ng/mL (*p* = 1.00) after 24-h incubation and 5.51 ± 0.87 (*p* = 0.07) after 48 h.

After 24-h incubation, 8-OHdG concentration increased 6.5-fold after treatment with LD50 (*p* = 0.03) and 21.5-fold after treatment with LD100 (*p* < 0.001). At both doses, extending the incubation time to 48 h caused a decrease in 8-OHdG concentration after treatment compared with 24-h incubation: from 6.26 ± 0.47 to 1.68 ± 1.12 ng/mL for LD50 (*p* = 0.11) and from 33.45 ± 0.15 to 9.73 ± 2.23 ng/mL for LD100 (*p* < 0.001). However, although the concentration of 8-OHdG decreased after 48 h of incubation, this value was still higher than controls (*p* < 0.01).

## 3. Discussion

Interest continues to grow in the use of entomopathogens and their metabolites as means of controlling pest populations [60,61]. The cosmopolitan soil fungus *C. coronatus* is able to selectively attack a number of insect species [62], one of which is twax moth *G. mellonella*. However, while research has shown that exposure to sporulating colonies of *C. coronatus* resulted in the death of 90% *G. mellonella* larvae [3,9], the infection process remains poorly understood.

Our present findings confirm that seven fractions extracted from the post-incubation medium of *C. coronatus* have insecticidal activity. Of these, the most active fraction was Fraction 3 (FR3), which caused the death of larvae and adults after injection or topical application, and disturbances in metamorphosis. GC-MS analysis of FR3 indicated the presence of octanoic acid, a short fatty acid, in this fraction, which has been found to have insecticidal activity against various groups of insects [63]. More importantly, octanoic acid is harmless to bees; it is a major volatile component of royal jelly; its concentration is much lower in food for drones and worker larvae than for the queen [23,64]. Research indicates the repellent activity of this compounds and field studies in bee colonies confirmed that the compound may interfere with the process of cell invasion by the mite and provides protection to honeybee young queens against *Varroa destructor* [23]. Moreover, treatment with octanoic acid reduced autogrooming behavior significantly of virgin queen honeybees, *Apis mellifera* [65]. Based on this insecticidal activity and its lack of toxicity against humans and bees, this compound was chosen for further examination.

Literature data have found that 50% of adult *Drosophila melanogaster* were killed by administration of 36 μg octanoic acid, while 50% of *D. sechellia* adults were killed after 200 (for females) or 245 (for males) μg [66]. Octanoic acid also demonstrated anti-*Galleria* activity in the present study, with a similar potency as observed for *D. sechellia*: 72% mortality of adults was observed after treatment with 100 μg of octanoic acid.

The acute toxicity of a compound is measured as the calculated dose of a substance that is expected to cause the death of 50% of a defined experimental animal population, i.e., the LD50 value. The LD50 value of octanoic acid for *G. mellonella* larvae was about 11 μg/mg (11,000 mg/kg) and is, hence, regarded as out of scale according to the Globally Harmonized System of Classification and Labelling of Chemicals (GHS); i.e., it does not cause acute toxicity after topical application [67]. Among the tested insects, *Blattella germanica* (7 μg/mg) and *Periplaneta americana* (0.4 μg/mg) were more sensitive to octanoic acid application, while *D. sechelia, Myrmica rubra* and *M. rudiginosis* were more resistant [63]; however, these data were obtained after six hours of incubation.

The LD50 values obtained in the present paper were similar to those obtained after dermal application of octanoic acid to mammalian model organisms: rabbits (LD50 > 5000 mg/kg) [68] and rats (LD50 >2000 mg/kg) [69]; this result supports the thesis that *G. mellonella* larvae might be useful tools to determine the toxicity of food additives.

The concentration of octanoic acid found is royal jelly is estimated as 1µg per 10 mg of royal jelly [23]. In the present study, each larva which received the LD50 dose and was treated with the same amount of octanoic acid, which is present in 20 mg of royal jelly.

During their evolution insects acquired two mains defense mechanisms against pathogens: the cuticle and the immune system. The epicuticle, the outermost surface of the insect cuticle, plays a key role in protecting insects against fungal infection [8,70,71]. The layer comprises various lipids, proteins, and phenolic compounds. These have been found to influence the rate of fungal growth and whether an attacking fungus develops. Beneath the epicuticle is a procuticle, consisting mainly of proteins and chitin [6,72], which is itself covered by a layer of saturated and unsaturated hydrocarbons, fatty acids, esters, alcohols, sterols, and aldehydes [72]; however, the profile of this layer varies between species, integument position, and developmental stage [73,74,75,76,77,78,79,80]. Due to its ambivalent properties, such as the hydrophilic part of carbonyl group and hydrophobic part of linear saturated carbon chain, octanoic acid is well suited to penetrate the insect cuticle [63]. However, research suggests that rearrangements in the insect cuticle may reduce the penetration ability of octanoic acid [66].

Membrane fluidity is influenced by its ratio of unsaturated to saturated fatty acids. In general, an increase in saturated fatty acid content promotes membrane stability, while cis-unsaturated fats reduce it. Longer chain lengths can provide greater stability and rigidity [81]. Adding exogenous oleic acid (C18:1) to the yeast media was shown to mitigate the toxic effects of octanoic acid [82,83].

The differences in LD50/LD100 observed for *G. mellonella* larvae and adults may be influenced by their variation in cuticle structure. In the present study, treatment with LD50 octanoic acid in larvae resulted in elevated cuticular FFA concentration, with an 898-times higher concentration of C8:0 being noted on the cuticle surface; this might suggest elevated C8:0 synthesis and/or transportation to the cuticle or, more likely, inefficient penetration of the topically applied tested compound through the cuticle. Interestingly, the level of C8:0 on the surface of the cuticle after administration of the LD50 dose was also higher than observed after LD100 treatment, and both were higher than in controls. While C8:0 levels were also elevated after LD50 and LD100 treatment (12- and 14-fold, respectively) in adults, this increase was not as large as in larvae.

The differences in LD100 value between larvae and adults are probably associated with changes in cuticle arrangement occurring during exposure. The huge accumulation of octanoic acid on the larval cuticle after treatment with the LD50 dose, i.e., 898-times greater than in control larvae, might be due to changes in the ratio in saturated/unsaturated FFAs. The predominant FFAs were C16:0, C18:2, C18:1, and C18:0 in control larvae, but C16:0, C18:2 (or C18:1 after treatment with LD100), C12:0, and C14:0, are predominant after octanoic acid treatment. In addition, the greatest increase in concentration following LD50 treatment was observed for the saturated FFAs C12:0, C14:0, and C15:0. Such higher saturated fatty acid concentrations might increase membrane sealing, thus lowering cuticle penetration by octanoic acid, which may be the reason for the huge accumulation of octanoic acid on the cuticle surface. In contrast, an increase in unsaturated FFAs (for example C16:1) was observed after treatment with the LD100 dose; this would facilitate the penetration of octanoic acid into the body of *G. mellonella*.

The application of LD50 to adults shifts the ratio of saturated/unsaturated FFAs by increasing the concentration of unsaturated FFAs: C16:0, C18:2, C18:1, and C18:0 predominated in controls, while C16:1, C18:2, and C18:1 predominated after LD50 treatment. Interestingly, the opposite changes in cuticular FFA concentrations were observed between larvae and adults, i.e., after applications of LD50, C9:0, C10:0, and C12:0 concentrations increased compared to controls in larvae, but decreased in adults. These changes might be responsible for the better penetration of octanoic acid through the cuticle and might account for the fact that the LD100 value is lower for adults than for larvae.

A key consideration in the topical application procedure is the choice of solvent. Acetone is believed to possess relatively low acute toxicity for mammals and for aquatic and terrestrial arthropods and is frequently used in physiological and biochemical investigations on insects [84]. Acetone is metabolized by carboxylation to acetoacetate, as shown in bacteria, and is incorporated into fatty acids during lipogenesis in mammal adipose tissue [85,86,87]; therefore, it is possible that the increase in concentration in some FFAs observed after acetone treatment may be due to acetone metabolism. However, this thesis needs further study. Presently, no data exist on the metabolism of acetone in insects or the presence of any enzymatic machinery that can convert acetone to FFAs in insect tissues. Insects have evolved a highly active immune system that is able to effectively kill or immobilise pathogens [88]. It consists of a series of interconnected humoral and cellular responses [89,90]. For example, the cellular immune response is mediated by haemocytes; these are cells that are able to phagocytose microbes and encapsulate larger objects such as wasp eggs [91,92].

The increase in cuticular FFA concentration noted following octanoic acid treatment may have been associated with the destruction of the fat body. Such damage would have resulted in the release of FFAs from host cells such as hemocytes; indeed, octanoic acid is known to destroy *G. mellonella* hemocytes and Sf-9 cells. It is possible that lipid metabolism is governed by different mechanisms depending on the stage of development, as indicated by the variable effects of octanoic acid treatment, and acetone, on the cuticle FFA profiles of larval and adults.

The homogenate from the entomopathogenic fungus, *C. coronatus* demonstrated toxic activity against Sf-9 cells (WST-1 test) [13]; in addition, the application of dodecanol, another metabolite of *C. coronatus*, demonstrated high toxicity against various insect cells and inhibited their growth [10].

Our findings indicate that octanoic acid can disturb mitochondrial activity in Sf-9 cells at lower concentrations than those needed to influence cell membrane structure. This may not only suggest that the mitochondria are more sensitive to octanoic acid but could also provide an insight into the potential mechanism of octanoic acid action against insect cells. The fact that a lower LC50 value was obtained with the WST-1 test suggests a loss of mitochondrial activity and function and, thus, cell function. It is important to mention that mitochondria serve several important functions, not only energy production, but also the management of apoptosis and oxidative stress [93,94]. Both of the latter processes have a destructive impact on the cell membrane, and cell membrane disturbances were also detected in of Sf-9 cells after octanoic acid treatment.

Therefore, this result, combined with the higher LC50 value obtained by the LDH test, might suggest that oxidative stress and/or apoptosis plays a role in cell death after octanoic acid treatment. Dose-dependent apoptosis-related changes in Sf-9 mitochondria were observed after treatment with abamectin, Vip3Aa, a soluble protein produced by certain *Bacillus thuringiensis* strains, as well as actinomycin-D, azadirachtin, harmine, and various extracellular organic compounds from the ichthyotoxic red tide alga *Heterosigma akashiwo* [94,95,96,97,98,99]. Despite this, the lower concentrations of applied octanoic acid were found to decrease the amount of LDH released from the cells in the present study, which might suggest that such low concentrations reduced membrane permeability, this being a potential cellular defense mechanism. Elevated levels of released LDH, as observed after treatment of Sf-9 cells with *Bacillus thuringiensis* Cry protein, are characteristic of necrotic cells [100]. This might suggest that the death of SF-9 cells after treatment with octanoic acid is caused by mitochondrial failure and as a result of apoptosis (low C8:0 concentration) and/or necrosis (high C8:0 concentration).

Our findings indicate that the cuticle, the first line of defense against infection, acts as a defense mechanism against the toxic effects of *C. coronatus* metabolites. However, research has shown that the enzyme cocktail produced by the fungus results in a higher rate of cuticle degradation in *G. mellonella* larvae, compared to resistant insects [3,79,101]. Moreover, high phagocytic and encapsulation activity, reduced phenoloxidase activity, and greater lysozyme-like activity have been observed in *G. mellonella* larvae after fungal exposure [102], which suggests that it is the immunological system of the wax month, not the exoskeleton, that plays a pivotal role against fungal infection.

*C. coronatus* has developed several mechanisms of killing *G. mellonella* immunocompetent cells, such as inducing oxidative stress [52] and apoptosis/necrosis [36] in hemocytes during infection. Similarly, the application of fatty acids has been found to induce oxidative stress, dysfunctional regulation of internal turgor pressure, lipid peroxidation, membrane trafficking, and the disruption of mitochondrial, vacuolar, and plasma membrane organization in these cells [103,104,105,106]. It has also been proposed that the modification of membrane rigidity activates the cellular stress response mechanism, leading to increased proton efflux, diminished membrane damage, and lower levels of ROS [107].

The production of ROS in *G. mellonella* is still unclear; however, it seems to be related to melanization via the activation of the phenoloxidase cascade. Enzymatic redox processes acting on the quinones produced in this cascade will generate superoxide an-ion and, subsequently, other ROS types. The activation of phenoloxidase can occur in different ways, depending on the type of infecting microorganism, which could result in different responses in the production of ROS. However, no specific studies have been performed to correlate these pathways with the type of pathogenic microorganism [46,59,108].

One of the consequences of cellular oxidative stress is DNA damage, which is commonly measured using 8-hydroxy-2-deoxyguanosine (8-OHdG) as a biomarker. Previous studies have indicated elevated 8-OHdG levels in *G. mellonella* larva hemolymph during infection with *C. coronatus* [52]. Our present findings also indicate that treatment with a fungal metabolite, viz., octanoic acid, results in changes in 8-OHdG concentration: Its level first increases and then decreases after 48 h of incubation. In contrast, in the case of fungal infection, higher levels were observed after both 24- and 48-h of incubation. These differences might be connected with lipoic acid activity. Lipoic acid is a well-known antioxidant which prevents oxidative damage in cells by acting as a cellular modulator, scavenging free radicals and inducing and regenerating endogenous antioxidant factors [109,110]. It has been found to counteract the toxicity of paraquat treatment in *D. melanogaster* [111,112]. It is possible that lipoic acid biosynthesis is stimulated by treatment with octanoic acid, which is involved in lipoic acid biosynthesis [113]. However, more research is needed to confirm this thesis.

The presence of oxidative stress in *G. mellonella* hemocytes after octanoic acid treatment might be connected also with activity of lipoxygenases: enzymes involved in fatty acid metabolism and contributed to formation of oxidative stress in cells. These enzymes might be activated in response to the presence of octanoic acid in cells, as well as by lipid peroxidation. Research on *Spodoptera litura* indicated that application of destruxin from the entomopathogenic fungus *Metarhizium anisopliae* induces oxidative stress. The authors note a considerable rise in lipoxygenase activity and lipid peroxidation in treated larvae, and this rose with a time of exposure to mycotoxin [114]. In contrast, however, it has been proposed that DNA damage may play a greater role in the death of *G. mellonella* hemocytes rather than lipid peroxidation after *C. coronatus* infection [52].

One of the consequences of oxidative stress is the occurrence of apoptosis. It is possible that apoptosis might be initiated by fatty acids, for example, the effect of palmitic and stearic acids on coronary endothelial cells [115] and HepG2 hepatocyte cells [116]. Eighteen fatty acids, both saturated and unsaturated, have been found to have pro-apoptotic effects in Jurkat (T-lymphoblastic leukemia) and Raji (Burkitt’s lymphoma cells) cell lines, with the pro-apoptotic properties depending on the length of the chain; i.e., longer acids have greater activity at lower concentrations and in the presence of unsaturated bonds in the chain, and unsaturated acids induce apoptosis more effectively that the saturated acids [117]. Studies have shown that a mixture of oleic and stearic acids (2:1) added to cultures of human liver cells (L-02) at a concentration of 1 mM leads to the occurrence of oxidative stress [118].

The most commonly described data example in the literature of apoptosis occurrence in Lepidoptera is that of insect metamorphosis, where during the larva–pupa transition the larval midgut epithelium of is progressively displaced by a new epithelial layer that grows underneath it during the larva–pupa transition; as a result, the larval midgut is pushed into the gut lumen, where it forms the yellow body, a compact mass of cells that subsequently die [119]. However, apoptosis also plays an important role in preventing infection in insects [120]. Studies on baculoviral infection suggest that apoptosis can be an extremely powerful response to infection, reducing viral replication, infectivity, and the ability of the virus to spread within the insect host even if successful infection is established. Apoptosis is especially effective when it is combined with other innate antiviral defenses [121,122,123]. Most previous literature data studying the role of apoptosis in the defense of insects against bacterial infections concern the mechanisms utilized by pathogens to repress the host apoptotic response [124,125]. For example, silencing the locust inhibitor of apoptosis protein 1 (LmIAP1) gene results in greater direct mortality and increased insect susceptibility to *Metarhizium acridum*, a locust-specific fungal pathogen. In turn, *Metarhizium anisopliae* infection has been found to induce fatal apoptosis in *Aedes aegypti* larvae; this process appears to depend on caspase activity, which is regulated by heat shock protein 70 (HSP 70) and inhibited by protease inhibitors [126]. Our previous studies with *G. mellonella* larvae infected with *C. coronatus* identified elevated levels of HSP 90, 60, and 27 in insect hemolymph but not of HSP 70, which suggests that the process of fungus-induced apoptosis is associated with other factors than HSP 70, at least in *G. mellonella* [127].

A central role in apoptosis is played by caspases: evolutionarily conserved cyste-ine-dependent aspartate-specific proteases whose role in mammals is well known and widely described. A proapoptotic proteolytic cascade takes place in which initiator caspases (caspases 2, 8, 9, and 10) are first activated are activated first in the pro-apoptotic proteolytic cascade, and these in turn activate effector caspases 3, 6, and 7 [128]. However, little information exists about the caspases in Lepidoptera, with only six such proteins and/or their genes being described: Sf-caspase-1 (*S. frugipedia*), Sl-caspase-1 (*S. littoralis*), Hearm caspase-1 (*H. armiger*), Gm-caspase-1 (*G. mellonella*), Cs-caspase-1 (*Chilo suppressalis*), and Bm-caspase-1 (*Bombyx mori*) [129,130,131]. Phylogenetic analyses have found Lepidoptera to possess at least six caspases; of these, Lep-Caspase-1, -2, and -3 are believed to be potential effector caspases, and Lep-Caspase-5 and -6 to be initiators, while the function of Lep-Caspase-4 remains unclear [132]. In contrast, seven caspases have been characterized in *D. melanogaster*: three initiators and four effectors [133]. Our present findings indicate that octanoic acid causes apoptosis in hemocytes, as was clearly seen after both treatment of tested compound: in vitro application of octanoic acid to the hemocyte culture and in haemocyte culture from larvae, in which octanoic acid was provided topically. However, the apoptosis process may demonstrate different dynamics than after in vitro and in vitro application: Changes occurred later in the cells treated in vivo than those treated in vitro. Moreover, the intensity of fluorescence for both caspase and apoptosis occurrence was lower than after direct treatment of hemocytes with octanoic acid. This result might be caused by the reduced permeability of the cuticle, resulting in the considerable accumulation of octanoic acid on its surface and might point also to the role of cuticle as a pivotal defense mechanism against octanoic acid.

## 4. Materials and Methods

### 4.1. Chemical Reagents

Octanoic acid (>99%, liquid) was obtained from Sigma Aldrich (product number C2875; CAS Number 124-07-2, St. Louis, MO, USA). The compound was dissolved in acetone (Avantor) or ethanol (Chempur) and given to *G. mellonella* topically (in vivo condition) or directly to hemocyte or Sf-9 cell culture (in vitro condition).

### 4.2. Insects

A culture of the wax moth, *G. mellonella*, was reared. The culture was maintained in temperature and humidity-controlled chambers (30 °C, 70% r.h.) in constant darkness on an artificial diet [134]. Three-day-old last instar larvae and three-day-old adults were used for testing the insecticidal activity of *C. coronatus* fractions and octanoic acid.

The larvae (last larval instar) and adult (three days old) were used for the in vivo analysis of octanoic acid application; in these cases, the compound was given topically at doses of LD50 and LD100 (lethal dose 50 and lethal dose 100). Two controls were used: untreated insects (C1) and insects that received acetone (larvae) or ethanol (adults) (C2). After treatment, the insects were incubated for 24 h under optimal conditions for growing. After incubation, they were counted, weighed, and either frozen at −70 °C for GC-MS analysis or hemolymph was collected for the detection of changes in hemocytes

#### Hemolymph Collection

The hemocyte cultures were established from freshly collected hemolymph of *G. mellonella*. The last instar larvae (wandering stage) were used in all experiments. Before bleeding, the larvae were washed with distilled water and then immersed briefly in 70% (*v*/*v*) ethanol to sterilize their surfaces. Hemolymph was collected from small incisions in the last proleg. One drop of hemolymph (26 µL) contained circa 1.3 × 10^5^ cells. The fresh hemolymph (six drops) was mixed with 300 μL of Grace Insect Medium (GIM; Gibco) supplemented with gentamycin (10 mg/mL; Gibco), amphotericin B (250 µg/mL; Gibco) and phenylthiourea (0.1 mM; PTU; Merck Millipore). The freely dripping hemolymph from insects was collected into sterile polypropylene 1.5 mL centrifuge tubes.

To detect the changes occurring in the *G. mellonella* hemocytes following in vitro application, 1 µL of octanoic acid was added to 24-h hemocyte cultures, to a final concentration of 0.33 and 0.16 µg/ µL respectively. Two controls were used: untreated cells (C1) and cells cultured with the addition of 1 µL of 99.9% ethanol (C2), to exclude the negative influence of the solvent. All samples were incubated in a Heratherm IMH 100-S incubator (Thermo Fisher, Waltham, MA, USA) at 27 °C and 80–90% humidity for 24 or 48 h. The collected hemolymph was used to detect changes in hemocyte morphology, and to detect apoptosis and caspase activity after in vivo and in vivo application of octanoic acid.

In both the in vivo and in vivo experiments, any morphological changes occurring after octanoic acid administration were recorded using an AxioVert A1 phase contrast fluorescence microscope with an AxioCam ICC5 camera and Zen 12 software (Zeiss); the same apparatus was used to register the occurrence of apoptosis and the caspase activity in hemocytes.

### 4.3. Fungus

*C. coronatus* (isolate no. 3491), originally isolated from *Dendrolaelaps* spp. (Mesostigmata: Digamasellidae), was obtained from the collection of Professor Bałazy (Polish Academy of Sciences, Research Centre for Agricultural and Forest Environment, Poznań, Poland). It was routinely maintained in 90 mm Petri dishes at 20 °C in an LD 12:12 h cycle to stimulate sporulation [135] on Sabouraud agar medium (SAM). To enhance the sporulation and virulence of the *C. coronatus* SAM cultures, the medium was supplemented with homogenized *G. mellonella* larvae at a final concentration of 10% wet weight.

Cultures aged seven days were rinsed in sterile water to harvest the conidia, and 100 µL portions of the suspension, each containing approximately 50 conidia, were used for the inoculation of 250 mL Luria Broth (LB) medium. In order to obtain *C. coronatus* metabolites fungus was cultivated without shaking at 20 °C in 80 Erlenmeyer flasks each containing 250 mL of LB. At four weeks after inoculation, the mycelia were removed by filtration through Whatman no. 1 filter paper.

### 4.4. Extractions Method

#### 4.4.1. Extraction of *C. coronatus* Filtrate

The obtained filtrate (20 L) was divided into 2.5 L portions, which were placed in porcelain 5 L beakers. Each portion was extracted three times at room temperature in a reactor for 15 min with 500 mL ethyl acetate (Avantar) with intense stirring. The layers were separated in a separating funnel. The ethyl acetate layers were combined and the solvent was evaporated in vacuo (Buchi rotary evaporator) to yield 4 g of oil. The crude extract dissolved in 5 mL of ethyl acetate was applied to a silica gel column (40 g, 70–240 mesh, Merck); the products were eluted first with hexane (60 mL) and then with hexane-ethyl acetate mixtures from 5:1 to 1:5 (each mixture 150 mL), ethyl acetate (100 mL), ethyl acetate-methanol 1:1 (100 mL), and methanol (300 mL). Solvents from seven collected fractions were evaporated in vacuo, and the insecticidal activity of obtained extracts was tested. Each fraction was weighed, dissolved in acetone, and portioned. Part of each fraction was used to detect the metabolites in filtrates by GC/MS and to identify any insecticidal activity against *G. mellonella* larvae and adults.

#### 4.4.2. Extraction of Free Fatty Acids (FFAs)

The cuticular lipid components of the insects were extracted, separated, and analyzed by GC–MS. The method of extraction was based on literature data [3,79,80,136,137]. Lipids from larvae and adults were extracted in 20 mL of dichloromethane (Merck Millipore) for 5 min to yield cuticular lipids. The extracts were placed in glass flasks and evaporated under nitrogen.

### 4.5. GC-MS Analysis

#### 4.5.1. Derivatization Method

One mg of the extracted fraction was silylated with 100 μL of N,O-Bis(trimethylsilyl) trifluoroacetamide (BSTFA): chlorotrimethylsilane (TMCS) (99:1) (Merck Millipore) mixture for one hour at 100 °C to obtain trimethylsilyl (TMS) esters. The cuticle samples were subjected to similar conditions, i.e., one mg of each sample and 10 µL of internal standard (19-methylarachidic acid; 1 mg/mL; Merck Millipore) were silylated with 100 μL BSTFA:TMCS (99:1) mixture for one hour at 100 °C to obtain trimethylsilyl esters (TMS) of FFAs. The TMS values of the fractions components and fatty acids were then analyzed by GC–MS; however, because it is well separated from all constituents of the sample and was not previously present in the insect samples, 19-methylarachidic acid was used as an internal standard (IS) in the GC analysis used for FFAs [138].

#### 4.5.2. GC-MS Analysis

The GC–MS analyses were carried out on a GCMS-QP2010 system with mass detector (Shimadzu, Kyoto, Japan). Helium was used as the carrier gas at a column head pressure of 65.2 kPa. For *C. coronatus* fractions analysis, a DB-5 MS (Zebron, Phenomenex, USA) column was used (thickness 0.25 µm, length 30 m, and diameter 0.25 µm). The column oven temperature cycle was maintained at 80 °C for 3 min, and then ramped from 80 to 310 °C at 4 °C/min; the final temperature was then held for 10 min. The FFA cuticular profile analysis was performed using a DB-5 MSi column (Zebron, Phenomenex, Torrance, CA, USA): thickness 0.25 µm, length 60 m, and diameter 0.25 µm. The column oven temperature cycle was maintained at 50 °C for 3 min, and then ramped from 50 to 310 °C at 4 °C/min; the final temperature was then held for 10 min. In both analyses, the ion source temperature was 200 °C and the interface temperature was 310 °C. Split mode was used with a split ratio of 10.

The trimethylsilyl ester of octanoic acid (C8:0) present in *C. coronatus* fractions and FFAs in the cuticular fractions were identified based on the fragmentation patterns and mass-to-charge ions of the TMS derivatives given in the NIST 11 library. The mass spectra of the fatty acid trimethylsilyl esters comprised M + (molecular ion), [M-15] + , and fragment ions at m/z 117, 129, 132, and 145. The GC analysis used 19-methylarachidic acid as an internal standard (IS). The composition of the analyzed samples was calculated based on the chromatogram peak areas. Each sample was analyzed in triplicate, and the results were expressed as means and standard deviation. The response factors of one were assumed for all constituents. The method is based on literature data [3,10,78,79,80,136,138,139].

### 4.6. The Evaluation of Insecticidal Activity of the Fractions Extracted from Fungal Filtrate and of Octanoic Acid against G. mellonella

Each fungal fraction was evaporated, and the extracts were dissolved in acetone, ethanol, or dimethyl sulfoxide (DMSO, Chempur). The fractions dissolved in acetone or ethanol were administrated by topical application to larvae and adults in a volume of 1 µL containing 2.5–250 µg/insect. Injections of extracts were made to *G. mellonella* larvae in a volume of 1 µL fraction dissolved in DMSO containing 2.5–50 µg/insect of each fraction. The condition of treated insects was observed for six days.

For intake with food, the fractions were dissolved in ethanol, and 5 mL of each fraction solution was incorporated into 4 g of insect food to achieve a final concentration of 0.5 or 1 mg/g of food, respectively. The solvent was then evaporated from the food at 35 °C in an oven for 48 h. Before the addition of fraction, the food was baked at 180 °C for one hour to dry it and to kill any microorganisms, which might be pathogenic for the insects. The food was prepared once for the entire experiment. The 10 larvae for each repetition consumed the treated food throughout the entire experiment. The control group consisted of insects fed with food treated with solvent (ethanol) instead of fractions. The condition of insects was observed for four days to establish the mortality rates and the further development of survivors. The experiment was conducted in three repetitions.

All fractions were given topically, by injection, and by dietary application to three-day-old final (seventh) instar larvae. At this stage, the larvae are still feeding: the physiological cessation of feeding prior to metamorphosis takes place on the fifth to the sixth day of the final instar [22].

Each experiment was performed in three repetitions. The number of insects used in each experiment is presented in Table S1.

Octanoic acid was directly administered to the dorsal side of the insect cuticle by topical application. Each insect received 5μL of octanoic acid solution containing 50 µg or 100 µg of tested compound and acetone as a solvent (dose based on literature data [66]). Each dose was tested on twenty insects in three repetitions. The treated insects were observed for twenty days and following parameters were observed every day: mortality rates, pupation, and adult eclosion among survivors. Two controls were used: untreated larvae (C1) and larvae topically applied with solvent—acetone (C2).

### 4.7. The Determination of the LD50 and LD100 Doses

Eleven doses of octanoic acid (3.35; 6.69; 10.05; 13.38; 17.50; 20.55; 23.60; 27.50; 31.30; 36.00 and 39.46 µg of octanoic acid per 1 mg of *G. mellonella* body mass) were administrated topically to larvae (acetone was used as a solvent) and adults (ethanol as a solvent). The larvae and adults were individually weighed; the experiments included larvae with a weight of about 200 mg and adults with about 60 mg. Untreated insects (C1) and insects treated topically with solvent (C2) were used as controls. The experiment was conducted in triplicate with each group containing 10 insects. The numbers of surviving insects in each group were counted after 24 and 48 h, and the percentage of surviving insects was calculated. The LD50 and LD100 doses of octanoic acid were estimated using GraphPad software.

### 4.8. The Determination of Morphological and Viability Changes of Sf-9 Cell Cultures after In Vitro Octanoic Acid Treatment

The Sf-9 cell line (Thermo Fisher Scientific, Waltham, MA, USA) was derived from the pupal ovarian tissue of the fall army worm, *Spodoptera frugiperda* (Lepidoptera). The Sf-9 cells were cultured in optimal growth conditions at 27° C in the appropriate Graces Modified Medium: TNM-FH (200 mL Gibco™ Grace’s Insect Medium Supplement (GIM, Thermo Fisher Scientific, Waltham, MA, USA)), 20 mL Fetal Bovine Serum (FBS, Thermo Fisher Scientific, Waltham, MA, USA), 400 μL (10 mg/mL) gentamycin (Sigma), and 400 μL (250 μg/mL) amphotericin B (Sigma). Passages were performed every five to seven days according to the manufacturer’s recommendations.

Sf-9 cells were added with 100 μL medium to the wells of a 96-well culture plate (Nest) at a concentration of approximately 20 × 10^3^ cells per well. Octanoic acid was dissolved in 99.9% ethanol, and each well-received 1μL of octanoic acid solution to obtain a final concentration of 0.60, 0.30, 0.150, 0.075, 0.038, 0.019, 0.009, or 0.005 μg/μL in each well. Two controls were used: untreated cells (C1) and cells treated with 99.9% ethanol (1μL, C2). The cells were incubated for 24 and 48 h.

Morphological changes were observed using the Olympus (type IX 50) inverted phase-contrast microscope with Color View (IIIu) camera connected with Cell D software.

#### 4.8.1. The Determination of Sf-9 Cell Viability

The cytotoxicity of octanoic acid was determined by calculating cell viability using Cell Proliferation Reagent WST-1 (Roche) and Cytotoxicity Detection Kit (LDH, Roche).

Sf-9 cells were grown for 24 or 48 h with octanoic acid in a 96-well tissue culture plate. Next, they were incubated with the WST-1 reagent (30 µL of WST-1 to each well) for four hours. After this, the absorbance was read at 440 and 650 nm in a BioTek HT spectrophotometer, and the results were calculated according to the manufacturer’s instructions.

The Cytotoxicity Detection Kit was used to detects the concentration of lactate dehydrogenase (LDH). Firstly, the optimal number of cells was determined based on a serial dilution of Sf-9 cells (ranging from 0 to 4.6 × 10^3^ cells/100 μL) in a 96-well plate (Nest), with TNM-FH medium used as a blank for the tests. After overnight incubation under optimal conditions, Sf-9 cells from each dilution were divided into two groups. The first group was used to detect the amount of spontaneously released LDH; these cultures were supplemented with sterile distilled water (10 µL). The second group was used to determine the maximum amount of released LDH; in this case, the culture was supplemented with 10 μL of lysis buffer and then incubated for 45 min. After this time, 50 µL of medium from each well (from the first and second variant) was transferred to a separate plate. Following this, 50 μL of the reaction mixture (0.6 mL of the buffer combined with 11.4 mL of the substrate mixture) was added to each well and incubated for 30 min at room temperature in darkness. After 30 min, the reaction was stopped using the stop buffer supplied by the manufacturer in the kit. The viability was determined based on the difference between the absorbance value at 490 and 680 nm. This finding allowed the determination of the optimal Sf-9 cell density for further studies.

The next step was to determine whether octanoic acid causes lysis of the cell membrane and thus the death of Sf-9 cells. The assay was performed on a 96-well plate. Sf-9 cells were added to each well to bring the final concentration to 2 × 10^3^ in TNM-FH medium. The cells were incubated under optimal conditions, and after 24 h, 1 µL octanoic acid was added to the cells to give a final concentration of 2.00, 1.00, 0.50, 0.25, 0.13, or 0.06 μg/μL. These concentrations were higher than those used in the WST test, because preliminary research has shown that lower doses of octanoic acid had no effect on LDH release. Three controls were used: Cm-maximum LDH release, Cs-spontaneous enzyme release, and Cr-solvent control (1 µL 99.9% ethanol was added to the samples). After 24 or 48 h of incubation, 10 µL of lysis buffer was added to the maximum release (Cm) control and again the plate was transferred to the incubator for 45 min. The remainder of the procedure was similar to that used in determining the optimal number of cells. To determine LDH activity, the difference between the absorbance at 490 and 660 nm was calculated. The result was calculated using the following formula:

$$\%\;\mathrm{Cytotoxicity}\;\mathrm{of}\;\mathrm{Tested}\;\mathrm{Compound}=\frac{S\left(A490-A660\right)-\mathrm{Cs}\left(A490-A660\right)}{\mathrm{Cm}\left(A490-A660\right)-\mathrm{Cs}\left(A490-A660\right)}\times 100\%$$

where

S—test sample;
Cs—control of spontaneous release of cellular LDH;
Cm—control of the maximum release of LDH from the cell;
A490—absorbance value at 490 nm;
A660—absorbance value at 660 nm.

The result is presented as percent mortality assuming that the cells constituting the control of the maximum release of LDH constitute 100% of the dead cells.

#### 4.8.2. The Calculation of Lethal Concentration (LC50)

After incubating the cells with octanoic acid (24 and 48 h), the cytotoxicity of the compounds was determined using WST-1 and LDH assays. The samples were incubated for 24 or 48 h, after which the WST-1 assay or LDH assay was performed, and cell viability was determined. The obtained values were used to calculate the LC50 with GraphPad (Prism). Two controls were prepared for each test. The first was a solvent control, in which 1 µL of 99.9% ethanol was added to the culture instead of the test substance. The second control included untreated cells: the test compounds or solvent were replaced with the same amount of medium-TNM-FH.

### 4.9. Detection of Apoptosis after In Vivo and In Vitro Administration of Octanoic Acid

After 24 h of hemocyte culture (both after in vivo and in vitro treatment with octanoic acid), the apoptosis/necrosis assay was performed according to the GFP CERTIFIED^®^Apoptosis/Necrosis Detection Kit (Enzo Life Sciences) instructions. For this purpose, a reaction mixture containing 5 µL of Annexin V solution (Apoptosis Detection Reagent-Annexin V EnzoGold), 5 µL of necrosis detection factor (Necrosis Detection Reagent), and 500 µL of binding buffer was added to the culture (all solutions were included in the commercial kit). Cells were incubated with the mixture for 15 min and protected from light. After this time, the mixture was removed from the cells, and the hemocytes were washed 2 times in the binding buffer. The results were obtained using the fluorescence microscope.

### 4.10. The Detection and Measurement of Caspase Activity

Carboxyfluorescein MultiCaspase Activity Kit by Enzo Life Sciences was used to measure caspase activity in hemocytes cultured after octanoic acid applications in vitro or in vivo. The test was carried out in two variants: observation under a fluorescence microscope (to detect active caspase) and spectrofluorometric measurement (to measure the caspase activity).

To detect caspase activity in *G. mellonella* hemocytes, the following procedure was used in accordance with the manufacturer’s recommendations. First, 30X FAM-VAD-FMK substrate was added to the culture medium in a ratio of 1:30. The cells were incubated at 37 °C for one hour in the dark. The cell nuclei were stained with Hoechst staining (1.5 µL of Hoechst dye to the final concentration of 0.5% *v*/*v*) and the dead cells with propidium iodide (PI, 1.5 µL of PI (0.05% *v*/*v*) to the culture). The culture was incubated for 10 min. After incubation, the cells were washed twice in washing buffer.

Caspase concentration following in vivo octanoic acid administration was determined based on spectrofluorimetric changes. Briefly, 20 drops of hemolymph were collected to 100 µL of insect physiological saline (IPS; 150 mM NaCl, 5 mM KCl, and 1 mM CaCl_2_). Substrate 30X FAM-VAD-FMK was added to each test and control samples; the substrate to hemolymph ratio was 1:30. The samples were incubated at 37 °C for one hour, protected from light. After this time, the samples were washed twice in the washing buffer by centrifugation for 5 min at room temperature and 400× *g*. After the final rinse, the pellet was dissolved in 400 µL of IPS. Following this, 100 µL of the mixture was transferred to three wells on a black 96-well Assay Plates (Nest). Fluorescence was read with a spectrofluorometer at 490 nm (excitation) and 520 nm (emission).

### 4.11. The Changes in 8-Hydroxy-2’-Deoxyguanosine (8-OHdG) Concentration in G. mellonella Hemocytes after In Vivo Application of Octanoic Acid to Larvae

The changes in 8-OHdG concentration were detected after in vivo applications of octanoic acid to the larvae. Hemolymph was taken from 20 insects (20 drops), added to 100 µL of IPS, and then centrifuged (2700× *g*, at 4 °C, for 10 min). The supernatants were diluted 20 x in the diluent sample solution (included in the commercial kit; dilution selected experimentally). The test was performed using the DNA Damage ELISA kit (Enzo Life Science); the calibration curve was set up, and the samples were prepared in accordance with the manufacturer’s recommendations. Following this, 50 µL of prepared standards and samples was added to each well of the 8-OHdG immunoassay plate in triplicate, followed by 50 µL of diluted Anti-8-OHdG mouse antibody. The samples were incubated for one hour. After an hour, the wells were washed six times in the washing buffer. Following this, 100 µL diluted Anti-Mouse IgG: horseradish peroxidase (HRP) Conjugate antibody was added to each well and incubated for one hour. After incubation, the wells were washed six times, and 100 µL of 3,3′,5,5′-tetramethylbenzidine (TMB) substrate was added to each well and left for 15 min in the dark. After this time, 100 µL of stop solution was added to the samples, and the absorbance was read at 450 nm in a BioTek HT spectrophotometer. The 8-OHdG concentration in the samples was calculated based on the standard curve.

### 4.12. Statistics

The obtained results were tested using the Student’s *t*-test and ANOVA. Post hoc analysis was performed using Tukey’s HSD Test or Dunnett test, with the results being significant at *p* ≤ 0.05; STATISTICA (StatSoft Polska) software was used for the analysis. The normality of the data was tested using the Kolmogorov–Smirnov test. The curves for the surviving levels of insects and Sf-9 cells were fitted to a non-linear log-logistic regression model by using GraphPad Prism,v. 6.0 (GraphPad Software)

## 5. Conclusions

Octanoic acid appears to be a new insecticidal metabolite of the fungus *C. coronatus*, which demonstrated a destructive impact on *G. mellonella* larvae, adults, and in vitro cultures of hemocytes and Sf-9 cell line. Its high LD50 and LD100 values, exceeding the GHS scale, and the fact that it is approved as a food additive with well-documented insecticidal activity indicate that the compound offers potential as an important component in the design of new insecticides that are safe for humans and the environment.

In addition, the fact that the obtained LD50 value against *G. mellonella* is similar to that obtained in studies on mammals indicates that the wax moth may serve as a reliable model in bioscience. In addition, as a cheap and easy-to-breed model free from ethical problems, *G. mellonella* also offers great potential as a replacement for mammalian models.

## Figures and Tables

**Table 1:** Effect of octanoic acid on *G. mellonella* larva mortality and development.

Treatments	Dose	N	Percent of Surviving Larvae (% ± SD)	Mean Time to Pupation (Days after Octanoic Acid Application ± SD)	Percent of Pupation (% ± SD)	Mean Time to Emergence (Days after Octanoic Acid Application ± SD)	Percent of Adults (% ± SD)
**C1**	0 µg (0 µL)	65	95.33 ± 0.47 ^A^	5.09 ± 1.22	88.33 ± 6.24 ^A^ (92.69 ± 6.96 ^#^)	15.21 ± 0.97	49.67 ± 10.96 ^A^ (55.96 ± 9.81 ^##^)
**C2**	0 µg (5 µL)	65	95.67 ± 3.30 ^B^	4.83 ± 1.25	91.33 ± 5.19 ^B^ (95.43 ± 3.56 ^#^)	13.98 ± 1.52	44.67 ± 8.18 ^B^ (49.04 ± 8.91 ^##^)
**octanoic acid application**	50 µg (5 µL)	60	93.33 ± 6.24 ^C^	5.17 ± 0.85	81.67 ± 12.47 ^C^ (88.33 ± 16.50 ^#^)	16,00 ± 0.00	26.67 ± 6.24 (32.34 ± 3.23 ^##)^
	100 µg (5 µL)	60	28.33 ± 22.48 ^A,B,C^	3.07 ± 2.17	20.00 ± 18.71 ^A,B,C^ (65.91 ± 15,91 ^#^)	10.67 ± 7.54	11.67 ± 10.27 ^A,B^ (61,11 ± 5,56 ^##^)

For research, three-day-old last instar larvae were used. C1—untreated control; C2—acetone application; N—total number of individuals; SD—standards deviation; ^#^ as a 100% was counting the number of surviving larvae; ^##^ as a 100% was counting the number of pupae; ^A,B,C^ Statistically significant differences between control and insects exposed to acetone or octanoic acid (ANOVA, Test HSD Tukey, *p* < 0.05). See Table S2 for raw data.

**Table 2:** FFA content of the cuticular lipids extracted from *G. mellonella* (μg/g of insect body mass ± SD).

FFA	Larvae				Adults			
	C1	C2	Octanoic Acid		C1	C2	Octanoic Acid	
			LD50 11.45 ± 1.21 (µg/mg of Body Mass)	LD100 33.56 ± 2.57 (µg/mg of Body Mass)			LD50 11.40 ± 1.18 (µg/mg of Body mass)	LD100 18.33 ± 2.49 (µg/mg of Body Mass)
**C6:0**	0.18 ± 0.00	0.43 ± 0.05	0.62 ± 0.09	1.15 ± 0.62	0.32 ± 0.00 ^A,B^	0.10 ± 0.04 ^B,C^	0.08 ± 0.05 ^A,D^	0.36 ± 0.10 ^C,D^
**C8:0**	0.20 ± 0.00 ^a^	0.74 ± 0.07 ^b^	179.55 ± 16.79 ^a,b^	28.57 ± 12.32	0.55 ± 0.00 ^A,B^	0.68 ± 0.20	6.88 ± 0.47 ^A^	7.79 ± 0.83 ^B^
**C9:0**	0.29 ± 0.00	1.07 ± 0.16	2.18 ± 1.41	2.33 ± 0.75	6.02 ± 0.32 ^A,B^	4.19 ± 0.26 ^A,C^	0.51 ± 0.18 ^A^	0.64 ± 0.15 ^B,C^
**C10:1**	1.42 ± 0.00 ^a,b,c^	Nd ^a^	Nd ^b^	Nd ^c^	0.11 ± 0.00 ^A,B,C^	ND ^A^	ND ^B^	ND ^C^
**C10:0**	0.53 ± 0.00 ^a^	0.91 ± 0.02 ^b^	5.54 ± 1.75 ^a,b,c^	1.69 ± 0.48 ^c^	1.33 ± 0.08 ^A^	9.29 ± 1.05 ^A,B,C^	0.36 ± 0.04 ^B^	1.59 ± 0.32 ^C^
**C11:0**	nd	nd	1.99 ± 1.39	nd	29.68 ± 1.19 ^A^	60.69 ± 5.16 ^A,B^	24.22 ± 1.76 ^B^	30.03 ± 2.67 ^A,B^
**C12:0**	1.77 ± 0.06 ^a^	16.37 ± 0.40 ^b^	96.86 ± 9.08 ^a,b^	47.03 ± 20.49 ^a^	2.21 ± 0.06 ^A,B^	4.63 ± 0.46 ^A,C^	1.47 ± 0.10 ^C,D^	5.61 ± 0.61 ^B,D^
**C13:0**	nd ^a^	Nd ^b^	2.80 ± 1.57 ^a,b,c^	Nd ^c^	0.51 ± 0.07	1.23 ± 0.41	1.10 ± 0.22	0.65 ± 0.14
**C14:1**	1.08 ± 0.09 ^a^	2.64 ± 0.83 ^a,b,c^	Nd ^b^	Nd ^c^	0.59 ± 0.10 ^A,B,C^	ND ^A^	ND ^B^	ND ^C^
**C14:0**	7.82 ± 0.13 ^a^	54.80 ± 3.63 ^a^	129.69 ± 7.67 ^a,b^	31.01 ± 12.0 ^b^	5.70 ± 0.48 ^A^	25.70 ± 3.79 ^A,B^	7.80 ± 0.58 ^B^	13.93 ± 1.12 ^A^
**C15:0**	1.62 ± 0.07 ^a,b^	21.27 ± 3.47 ^a,c^	30.47 ± 4.22 ^b,d^	6.2 ± 2.711 ^c,d^	0.72 ± 0.11 ^A^	1.68 ± 0.32 ^B^	2.31 ± 0.19 ^A^	3.32 ± 0.49 ^A,B^
**C16:1**	3.23 ± 0.25 ^a^	14.89 ± 0.92	33.07 ± 10.52 ^a,b^	16.11 ± 2.29 ^b^	3.21 ± 0.53 ^A^	4.32 ± 0.92 ^B^	41.64 ± 6.60 ^A,B^	25.82 ± 6.09 ^A,B^
**C16:0**	99.04 ± 3.67 ^a,b,c^	1108.29 ± 183.53 ^a^	1139.12 ± 78.65 ^b^	541.71 ± 238.95 ^c^	68.19 ± 2.59 ^A^	42.03 ± 4.64 ^B^	53.03 ± 6.75 ^C^	110.21 ± 16.72 ^A,B,C^
**C17:1**	0.55 ± 0.03 ^a,b,c^	Nd ^a^	Nd ^b^	Nd ^c^	ND	ND	ND	ND
**C17:0**	1.55 ± 0.01	6.68 ± 2.87	9.68 ± 6.94	nd	0.60 ± 0.06 ^A,B,C^	ND ^A^	ND ^B^	ND ^C^
**C18:3**	3.45 ± 1.34	nd	nd	nd	3.82 ± 0.91 ^A,B,C^	ND ^A^	ND ^B^	ND ^C^
**C18:2**	84.90 ± 4.08 ^a,b,c^	nd^a^	130.56 ± 14.93 ^b^	Nd ^c^	77.55 ± 9.88 ^A,C^	ND ^A^	35.41 ± 16.56 ^A^	7.82 ± 5.57 ^C^
**C18:1**	72.61 ± 10.14	99.18 ± 31.09	57.42 ± 31.79	73.62 ± 37.08	46.15 ± 15.53 ^A,B^	10.50 ± 6.01 ^A^	12.08 ± 3.31 ^B^	27.96 ± 6.46
**C18:0**	15.70 ± 0.74 ^a,b,c^	Nd ^a^	Nd ^b^	Nd ^c^	22.09 ± 2.09 ^A,B^	4.62 ± 0.87 ^A^	1.34 ± 0.22 ^B^	7.72 ± 2.01 ^B^
**C20:1**	nd	nd	nd	nd	2.30 ± 0.10 ^A,B,C^	ND ^A^	ND ^B^	ND ^C^
**C20:0**	nd	nd	nd	nd	1.27 ± 0.04 ^A,B,C^	ND ^A^	ND ^B^	ND ^C^
**C24:0**	nd	nd	nd	nd	2.31 ± 0.54 ^A,B,C^	ND ^A^	ND ^B^	ND ^C^
**C26:0**	nd	nd	nd	nd	1.83 ± 0.40 ^A,B,C^	ND ^A^	ND ^B^	ND ^C^
**Sum of FFAs**	299.10 ± 18.03 ^a^	110.61 ± 13.37 ^b^	1820.25 ± 83.96 ^a,b,c^	745.11 ± 331.50 ^c^	277.04 ± 3.508 ^A^	169.65 ± 16.23 ^A^	181.26 ± 32.13	251.80 ± 40.66

C1—untreated larvae; C2—larvae that received 5 µL of acetone (larvae) or ethanol (adults); FFAs—free fatty acids; SD—standard deviation; nd/ND—not detected; statistically significant differences are marked with the same letters (small letters (^a,b,c,d^) were used for larvae and capital letters (^A,B,C,D^) for adults; ANOVA, Tukey’s HSD Test, *p* < 0.05); see Table S4 for raw data.

## Data Availability

All data generated or analyzed during this study are included in this published article and its supplementary information files.

## Supplementary Materials

The following supporting information can be downloaded at: https://www.mdpi.com/article/10.3390/ijms23095204/s1.
